# Self-Perceived Knowledge and Self-Reported Clinical Use of Minimally Invasive Endodontics Among General Dentists and Endodontists in Türkiye: A Cross-Sectional Survey

**DOI:** 10.3390/healthcare14193279

**Published:** 2026-10-02

**Authors:** Selin Bıçaklıoğlu, Melis Oya Ateş

**Affiliations:** Department of Endodontics, Faculty of Dentistry, Bolu Abant Izzet Baysal University, Bolu 14030, Türkiye; selin.bicaklioglu@ibu.edu.tr

**Keywords:** minimally invasive endodontics, minimally invasive access cavity, vital pulp therapies, minimally invasive root canal preparation, cross-sectional survey

## Abstract

**Highlights:**

**What are the main findings?**

- Endodontists reported higher self-perceived knowledge of minimally invasive endodontics (MIE) and more frequent self-reported clinical use of MIE approaches than general dentists.
- In multivariable models including professional group, professional experience, institutional setting, and weekly number of root canal treatments, professional group remained associated with the reported use of vital pulp therapies and minimally invasive root canal preparation, but not with minimally invasive access cavity use.

**What are the implications of the main findings?**

- The observed differences in perceived training adequacy and self-reported clinical use of MIE identify potential areas in which additional educational support may be beneficial.
- Greater emphasis on practical MIE education and clinical support may facilitate wider adoption of MIE in routine practice.

**Abstract:**

**Background/Objectives**: Minimally invasive endodontics (MIE) has gained increasing attention as an approach focused on preserving dental tissues while achieving the objectives of endodontic treatment. This study compared self-perceived knowledge, perceived adequacy of education, self-reported clinical use and practices, implementation difficulties, and barriers to routine use of MIE approaches among general dentists and endodontists in Türkiye. **Methods**: This online cross-sectional survey used non-probability voluntary sampling and included 300 participants (150 general dentists and 150 endodontists). Group comparisons used Pearson’s chi-square test, Yates-corrected Pearson’s chi-square test, or Fisher’s exact test as appropriate. For multiple-response items, option-specific comparisons were adjusted using the Benjamini–Hochberg false discovery rate (FDR) procedure. Associated factors were evaluated using binary logistic regression, with professional group, professional experience, institutional setting, and weekly number of root canal treatments included simultaneously in multivariable models. **Results**: Endodontists reported higher self-perceived knowledge of MIE and more frequent self-reported use of MIE approaches than general dentists (both *p* < 0.001). In multivariable models, endodontist status was associated with higher odds of reported use of vital pulp therapies (odds ratio (OR) = 5.626) and minimally invasive root canal preparation (OR = 2.238), whereas the association with minimally invasive access cavity use was not statistically significant (OR = 1.668; *p* = 0.200). General dentist status was associated with higher odds of reporting difficulties during vital pulp therapies (OR = 10.420) and minimally invasive root canal preparation (OR = 2.379), whereas no significant association was detected for minimally invasive access cavity difficulties. The use of magnification systems and specialized endodontic burs for access cavity preparation or refinement was reported more frequently among endodontists. Reduced loss of tooth structure was the most frequently selected reason for using MIE, while inadequate training or lack of knowledge was the most frequently reported barrier to routine implementation. **Conclusions**: Among the participating dentists, endodontists reported higher self-perceived MIE knowledge and more frequent self-reported clinical use than general dentists. Professional and practice-related characteristics were associated with several MIE-related outcomes. Greater emphasis on practical, case-based training may support the appropriate implementation of MIE; however, the findings should be interpreted in light of the cross-sectional, self-reported design.

## 1. Introduction

The primary goal of endodontic treatment is to prevent or treat diseases affecting the pulp and periapical tissues and to preserve the function of the affected tooth for as long as possible [1]. Achieving this goal requires the preparation of an appropriate access cavity to the root canal system, removal of infected or necrotic tissues, and effective cleaning and obturation of the root canal space [2,3]. However, the loss of hard dental tissues resulting from caries removal, removal of existing restorations, access cavity preparation, and root canal shaping may reduce the resistance of the remaining tooth structure [4,5,6]. Therefore, preservation of pericervical dentin has been suggested to contribute to the structural integrity of endodontically treated teeth, prompting a reassessment of traditional access cavity designs [7,8,9].

Minimally invasive endodontics (MIE) is a treatment philosophy based on preserving pulp vitality and dental hard tissues as much as possible without compromising the biological objectives of endodontic treatment [8,10,11]. This approach is not limited to the preparation of a smaller access cavity. Appropriate diagnosis and case selection, preservation of pulp vitality when indicated, limitation of unnecessary dentin removal during access cavity preparation, and root canal preparation in accordance with biological requirements are among the main clinical components of MIE. Magnification and imaging systems, together with irrigation activation methods, may be regarded not as direct MIE procedures themselves but rather as adjunctive methods that can facilitate the safe and effective implementation of these approaches in appropriately selected cases [12,13,14]. Current recommendations issued by the European Society of Endodontology (ESE) and the American Association of Endodontists (AAE) also recognize vital pulp therapies as valid treatment options in appropriately selected cases [1,15,16].

Nevertheless, it has not yet been conclusively demonstrated that minimally invasive access cavity designs provide better long-term clinical outcomes or higher tooth survival rates than traditional access cavity designs [11,17,18]. Although conservative access cavity designs may reduce coronal and pericervical dentin loss, excessive restriction of cavity dimensions may limit visibility and the operative field. This may make canal orifice identification, root canal shaping, irrigation, and obturation more difficult and may increase the risk of procedural complications [17,19]. Evidence regarding the effects of limited root canal enlargement on disinfection and irrigation efficacy also remains inconclusive [2,11,20]. Given that the available evidence is largely based on laboratory studies, that methodological heterogeneity exists among studies, and that long-term clinical data remain limited, the benefit–risk balance of MIE approaches should be evaluated according to the diagnostic, anatomical, and clinical characteristics of each case [11,17,18].

The translation of evidence-based knowledge into daily clinical practice is influenced by various individual and professional factors, including the clinician’s education, professional experience, clinical skills, and access to the necessary technical resources [21]. A recent systematic review evaluating studies on nonsurgical root canal treatment reported considerable variability in dentists’ knowledge levels, which were generally found to range from low to moderate. The same review also highlighted that educational approaches based solely on knowledge transfer may be insufficient to change clinical behavior [22]. These findings suggest that awareness of contemporary approaches in endodontics alone does not necessarily ensure their translation into clinical practice. A previous online study conducted in Türkiye reported that awareness of MIE was not reflected to the same extent in routine clinical practice [23]. However, the limited representation of endodontists in that study restricted comprehensive comparisons between general dentists and endodontists. Furthermore, more comprehensive data are needed regarding the perceived adequacy of MIE education, procedure-specific difficulties, the use of diagnostic and technical adjuncts, and perceived barriers to the adoption of MIE.

The aim of this study was to compare general dentists and endodontists practicing in Türkiye in terms of their self-perceived knowledge of MIE, perceived adequacy of the education they had received, preferred information sources, frequency of self-reported clinical use, and the reported use of minimally invasive access cavities, vital pulp therapies, and minimally invasive root canal preparation. The associations of professional group, professional experience, institutional setting, and weekly number of root canal treatments with the use of these three MIE approaches, the reporting of difficulties during their clinical application, and the use of magnification systems and specialized endodontic burs for access cavity preparation or refinement were also evaluated. Radiographic diagnostic methods, perceived barriers to the routine clinical implementation of MIE, and recommendations for promoting its wider adoption were additionally investigated.

## 2. Materials and Methods

### 2.1. Study Design and Ethical Approval

This study was an online, cross-sectional survey conducted to evaluate self-perceived knowledge levels, perceptions, and self-reported clinical use and practices related to MIE among general dentists and endodontists in Türkiye.

The study was conducted in accordance with the ethical principles of the Declaration of Helsinki [24] and was approved by the Non-Interventional Clinical Research Ethics Committee of Bolu Abant Izzet Baysal University on 26 August 2025 (Decision No. 2025/372). On the introductory page of the survey, participants were informed about the purpose of the study, the target participant group, the estimated time required to complete the survey, the voluntary nature of participation, and the fact that the collected data would be used solely for scientific purposes. Individuals who did not provide electronic informed consent to participate were not permitted to proceed with the survey. The study was reported in accordance with the Strengthening the Reporting of Observational Studies in Epidemiology (STROBE) guidelines [25], and the completed STROBE checklist is provided as Supplementary File S1.

### 2.2. Questionnaire Development and Assessment of Content Validity

The questionnaire was developed by the researchers in accordance with the study objectives and with consideration of the relevant literature on MIE [2,11,15]. The initial draft consisted of 23 questions, including six questions assessing participants’ demographic and professional characteristics and 17 questions related to MIE.

The content validity of the MIE-related section of the questionnaire was evaluated in two rounds by an expert panel comprising seven endodontists with at least five years of clinical and academic experience in the field of endodontics. For the content validity analysis, each questionnaire question was treated as a separate item. In the first round, the experts were asked to evaluate each question in terms of its relevance to MIE and the extent to which it represented the intended content using a four-point rating scale. The rating scale was defined as follows: 1 = not relevant, 2 = major revision required for the question to be considered relevant, 3 = relevant but requiring minor revision, and 4 = highly relevant [26]. The clarity and comprehensibility of the questions and the appropriateness of the response options were evaluated separately from the relevance ratings through written comments provided by the experts.

For each question, the item-level content validity index (I-CVI) was calculated as the proportion of experts who rated the question as 3 or 4 in terms of relevance. An I-CVI threshold of ≥0.78 was adopted; for a seven-member expert panel, questions receiving a positive rating from at least six experts were considered acceptable (I-CVI ≥ 0.86) [27].

In the first evaluation round, one questionnaire question that did not meet this criterion was removed entirely from the draft. Accordingly, the number of questions in the MIE section was reduced from 17 to 16, and the total number of questions in the questionnaire was reduced from 23 to 22. The response options for multiple-choice and multiple-response questions were also qualitatively evaluated in terms of comprehensiveness, clinical appropriateness, clarity, overlap among options, and potential logical inconsistencies. Based on the experts’ written comments, two questions were reworded, and the response options were revised where deemed necessary.

Following these modifications, the 16-question MIE section was submitted to the same expert panel for a second evaluation round. The scale-level content validity index/average (S-CVI/Ave) was calculated as the mean of the final I-CVI values obtained in the second round and was found to be 0.90 [26]. This result was considered supportive expert-based evidence of the content validity of the MIE section. The first six questions assessing demographic and professional characteristics were not included in the I-CVI or S-CVI/Ave calculations.

### 2.3. Pilot Study and Sample Size

A pilot study was conducted before the main data collection period to evaluate the comprehensibility of the final questionnaire, the appropriateness of the response options, the conditional skip logic, and its overall feasibility. The pilot study included a total of 30 participants, comprising 14 general dentists and 16 endodontists. The estimated time required to complete the questionnaire was approximately five minutes. Following the pilot study, no changes were deemed necessary to the content of the questions, the response options, or the conditional skip logic. Participants in the pilot study were not included in the analytical sample of the main study.

The minimum sample size required for the main study was calculated using G*Power version 3.1.9.7 (Heinrich Heine University Düsseldorf, Düsseldorf, Germany) based on effect sizes derived from the pilot study data. Based on chi-square comparisons conducted using the pilot data, Cohen’s *w* = 0.20, representing the lower end of the observed effect sizes, was adopted as a conservative effect size for the sample size calculation. With α = 0.05, 1 − β = 0.80, and 3 degrees of freedom, the minimum required sample size was calculated as 273. To account for potentially incomplete questionnaires or questionnaires that might need to be excluded from the analysis, the target sample size was increased by approximately 10% and set at 300. In addition, because binary logistic regression analyses were planned for selected MIE-related outcomes, an a priori sample-size analysis for binary logistic regression was performed before the main data collection using the large-sample z-test based on the Demidenko method with variance correction. The pilot study yielded an odds ratio (OR) of approximately 12 for the predictor-outcome relationship used for this calculation. Because effect estimates derived from a relatively small pilot sample may be imprecise and may overestimate the underlying effect magnitude, a priori sensitivity analyses were also performed using smaller hypothetical ORs of 9, 6, and 3. Assuming a two-sided α = 0.05, 95% power, Pr(Y = 1|X = 1) under H0 = 0.538, R^2^ = 0.507, representing the proportion of variance in the predictor explained by the remaining predictors, and a binomial predictor distribution with π = 0.533, the required total sample sizes were 135, 151, 192, and 413 for ORs of 12, 9, 6, and 3, respectively. Under these prespecified assumptions, the planned sample size of 300 provided at least 95% power for ORs of 6 or greater and approximately 86.5% power under the more conservative OR = 3 sensitivity scenario.

### 2.4. Participants, Sampling Method, and Data Collection

The target population consisted of general dentists and endodontists who were actively practicing in Türkiye during the data collection period. Eligibility criteria were presented on the introductory survey page, and participants electronically confirmed that they met these criteria before proceeding to the questionnaire. Eligibility was therefore based on participants’ self-declaration on the introductory survey page and their reported professional status as “General Dentist” or “Endodontist” in the questionnaire. Electronic informed consent was obtained from all participants before participation.

A non-probability, voluntary sampling method based on multichannel online invitations to the target participant population was used. Data for the main study were collected between 25 February and 25 May 2026 using Google Forms (Google LLC, Mountain View, CA, USA). The survey link was distributed through institutional email networks, emails sent by the Turkish Endodontic Society to its endodontist members, and professional WhatsApp groups for dentists.

No individual-level random sampling was performed, and no predetermined quota was applied to either professional group. The equal numbers of general dentists and endodontists in the final analytical sample occurred as a result of the participant recruitment process and were not predetermined. Because the distribution channels potentially overlapped and the number of eligible individuals who received or accessed the survey link could not be determined, neither the number of unique individuals invited nor the overall response rate could be calculated.

A total of 308 survey responses were received during the data collection period. Eight incomplete responses were excluded from the analysis, resulting in a final analytical sample of 300 participants. To restrict each participant to a single submission, the Google Forms “Limit to 1 response” setting was enabled, which required participants to sign in with their Google accounts. Because email address collection was disabled, participants’ email addresses were not recorded in the form responses accessible to the researchers or included in the analytical dataset. No directly identifiable participant information was collected in the research dataset.

### 2.5. Questionnaire Structure

The final questionnaire comprised two sections and a total of 22 questions. The first section consisted of six questions assessing participants’ demographic and professional characteristics. The second section contained 16 questions assessing self-perceived knowledge of MIE, perceived adequacy of MIE education, preferred sources of information, frequency of self-reported clinical use and other self-reported clinical practices, difficulties encountered during implementation, diagnostic and technical adjuncts used, and perceived barriers and recommendations. No standardized operational definition of MIE or of the individual MIE approaches was provided within the questionnaire. This approach was intended to preserve participants’ pre-existing understanding of these concepts, as providing standardized definitions immediately before the relevant questions could have introduced a common interpretation at the time of assessment. Participants therefore responded based on their pre-existing understanding of MIE and its individual approaches.

The questionnaire included single-response questions, multiple-response questions, and questions with conditional skip logic. An “Other” response option was provided for selected questions; however, no participant selected this option, and it is therefore not presented in the corresponding tables. An English translation of the original Turkish questionnaire administered to the participants is provided in Supplementary File S2.

### 2.6. Statistical Analysis

For conditional questions, participants who were not required to answer a particular question because of the survey skip logic were excluded from the denominator of the corresponding descriptive analysis unless otherwise specified. However, for the option-specific comparisons and the binary logistic regression analyses of the three specific MIE approaches listed in Question 11, participants who reported never using MIE in Question 10 were classified as non-users of each individual approach; therefore, these analyses were conducted using the full sample (*n* = 300). The presence of reported difficulties was evaluated only among participants who reported using the relevant MIE approach, whereas the procedural stages at which difficulties were encountered were evaluated only among participants who used the relevant approach and reported at least one difficulty. For conditionally displayed multiple-response items included in the option-specific between-group analyses, structurally skipped responses were not treated as item-missing data. Instead, each response option was coded as a participant-level binary indicator of whether that option was selected (1 = selected, 0 = not selected). Participants who were not presented with a conditional item because of their response to the preceding screening question were coded as 0 for the individual options of that item. Accordingly, the option-specific comparisons of reasons for preferring and not preferring MIE were conducted using the full professional-group samples (*n* = 150 per group). The resulting percentages therefore represent the proportion of all participants in each professional group who reported each reason, rather than the conditional distribution of reasons among MIE users or non-users only.

Data were analyzed using IBM SPSS Statistics, Version 23.0 (IBM Corp., Armonk, NY, USA). Categorical variables were summarized as frequencies (*n*) and valid percentages (%). For multiple-response items, each response option was coded as a separate participant-level binary variable (1 = selected, 0 = not selected) and analyzed separately using an option-specific 2 × 2 contingency table comparing general dentists and endodontists. No omnibus contingency-table test across the response options was performed. Pearson’s chi-square test, Yates-corrected Pearson’s chi-square test, or Fisher’s exact test with Monte Carlo simulation was applied as appropriate according to the cell frequencies. The Benjamini–Hochberg false discovery rate (FDR) procedure was applied within prespecified families of between-group categorical comparisons, including the option-specific comparisons for multiple-response items, as specified in the corresponding table footnotes. Both raw and FDR-adjusted *p* values were reported. For comparisons subject to FDR adjustment, statistical significance was determined based on an adj. *p* < 0.05; findings with a raw *p* < 0.05 but an adj. *p* ≥ 0.05 were not considered statistically significant. Effect sizes for these option-specific comparisons were expressed as Phi coefficients, with 95% confidence intervals calculated using the percentile bootstrap method based on 2000 resamples. For other categorical comparisons, Pearson’s chi-square test or Fisher’s exact test with Monte Carlo simulation was used as appropriate. Monte Carlo analyses were based on 5000 samples and a 95% confidence level. For statistically significant contingency tables with more than two categories, category-level differences in proportions were examined using Bonferroni-adjusted Z tests for column proportions. Effect sizes were expressed as Phi for 2 × 2 tables and Cramér’s V for larger contingency tables, with 95% confidence intervals calculated using the percentile bootstrap method based on 2000 resamples.

Binary logistic regression analyses were performed to evaluate variables associated with the use of minimally invasive access cavities, vital pulp therapies, and minimally invasive root canal preparation; the reporting of at least one difficulty during these approaches; and the use of magnification systems and specialized endodontic burs for access cavity preparation or refinement. Professional group, professional experience, institutional setting, and weekly number of root canal treatments were examined as explanatory variables. Separate univariable models were first constructed for each explanatory variable, followed by multivariable models including all four variables simultaneously. Age was not included simultaneously with professional experience because these variables represent closely related dimensions of career duration, and their concurrent inclusion could introduce redundant adjustment and potential multicollinearity. Professional experience was therefore retained as the indicator of cumulative clinical experience.

Because the effective sample size varied across the outcome-specific regression analyses, the precision and interpretability of the estimates were considered in relation to the available outcome information and model complexity. Particular caution was applied to models based on smaller conditional subsamples, and the precision of the estimated ORs was evaluated using their 95% confidence intervals (CIs). Model fit was assessed using the Hosmer–Lemeshow goodness-of-fit test, and Cox–Snell and Nagelkerke pseudo-R^2^ values were reported. Results are presented as odds ratios (ORs), 95% CIs, and *p* values. All tests were two-sided. For analyses not subject to FDR adjustment, a nominal *p* < 0.05 was used as the threshold for statistical significance. Because no single study-wide multiplicity adjustment was applied across all statistical analyses, findings from secondary and exploratory analyses, particularly those with nominal *p* values close to 0.05, were interpreted cautiously and in conjunction with effect estimates and 95% CIs.

## 3. Results

### 3.1. Demographic and Professional Characteristics of the Participants

A total of 300 participants, comprising 150 general dentists and 150 endodontists, were included in the analyses. Of the participants, 170 (56.7%) were female. The distribution of sex did not differ significantly between general dentists and endodontists (*p* = 0.244), whereas significant differences were observed in age, professional experience, institution of employment, and weekly number of root canal treatments (all *p* < 0.001).

At the category level, participants aged 23–30 years were more frequently represented among general dentists, whereas those aged 31–40 years were more frequently represented among endodontists. General dentists had higher proportions of participants with 0–5 and ≥16 years of professional experience, whereas the proportions with 6–10 and 11–15 years of experience were higher among endodontists. Employment at a university hospital was more frequent among endodontists, whereas employment at a public hospital/oral and dental health center or private practice/polyclinic was more frequent among general dentists. General dentists more frequently reported performing 0–5 or 6–10 root canal treatments per week, whereas endodontists more frequently reported performing 11–15, 16–20, or ≥21 treatments per week (Table 1).

### 3.2. Perceived Adequacy of MIE Training and Preferred Information Sources

The distribution of responses regarding the perceived adequacy of MIE training differed significantly between general dentists and endodontists (*p* = 0.002). The proportion of participants who considered their training adequate was higher among endodontists than among general dentists (20.0% vs. 11.3%). Similarly, endodontists more frequently considered their training partially adequate (56.7% vs. 45.3%), whereas general dentists more frequently considered their training inadequate (38.0% vs. 19.3%).

For the multiple-response item assessing preferred information sources, each response option was compared separately between professional groups, with FDR adjustment applied across the option-specific comparisons. Congresses, seminars, or courses (78.7% vs. 50.7%) and academic articles or publications (84.0% vs. 40.7%) were more frequently reported as preferred information sources by endodontists. In contrast, social media platforms (68.7% vs. 45.3%) and experience sharing with other dentists (62.0% vs. 34.7%) were more frequently reported by general dentists. Online education did not differ significantly between the professional groups after FDR adjustment (41.3% vs. 52.0%; adjusted *p* = 0.064) (Table 2).

### 3.3. Self-Perceived MIE Knowledge and Frequency of Self-Reported Clinical Use

**Figure 1:**
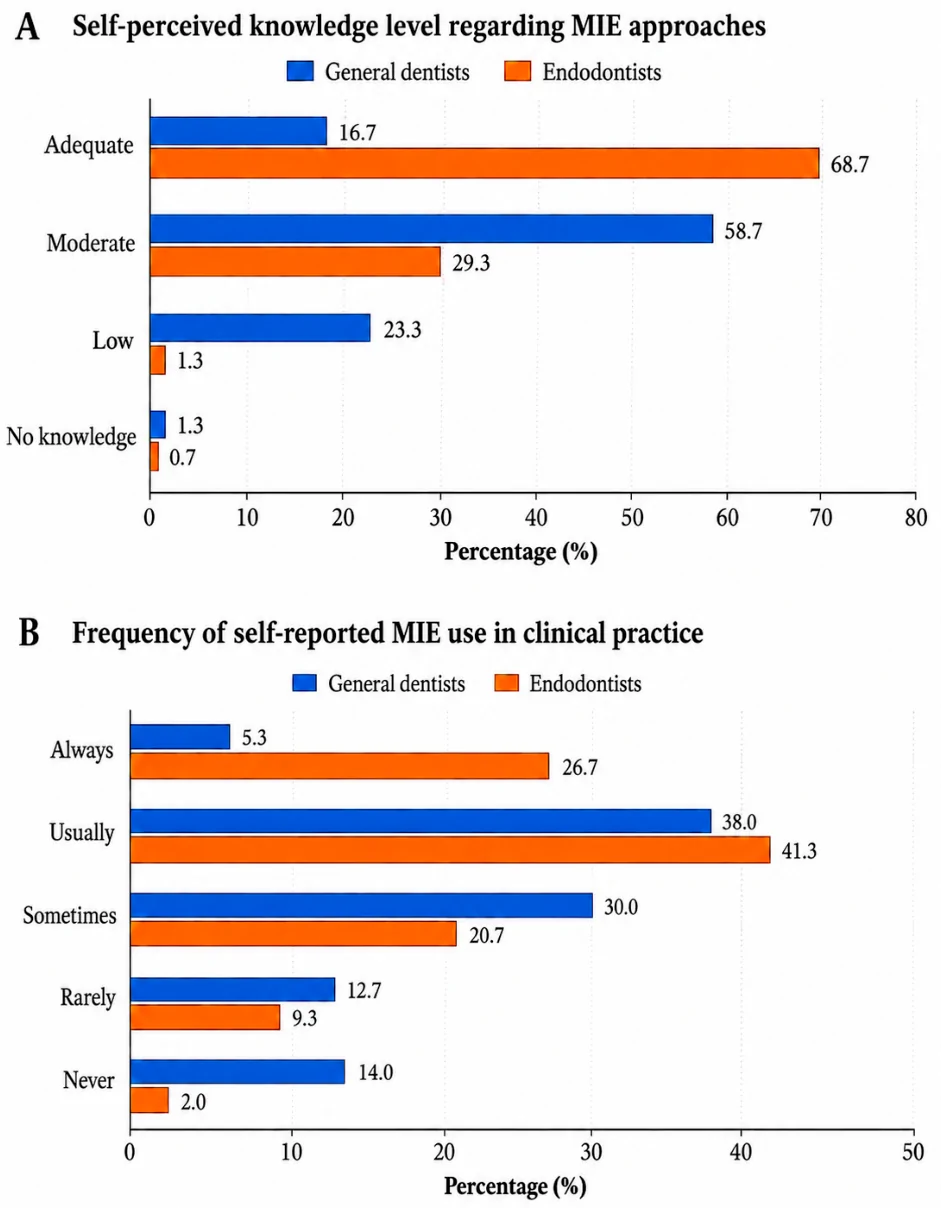
Self-perceived knowledge and self-reported clinical use of minimally invasive endodontics (MIE) among general dentists and endodontists. Values are presented as percentages.

The distribution of self-perceived MIE knowledge differed significantly between general dentists and endodontists (FDR-adjusted *p* < 0.001; Cramér’s V = 0.554, 95% CI: 0.445–0.669). An adequate level of knowledge was reported by 68.7% of endodontists and 16.7% of general dentists. Among general dentists, 58.7% reported a moderate level of knowledge and 23.3% reported a low level of knowledge, compared with 29.3% and 1.3% of endodontists, respectively (Figure 1A).

The frequency of self-reported MIE use in clinical practice also differed significantly between the professional groups (FDR-adjusted *p* < 0.001; Cramér’s V = 0.358, 95% CI: 0.255–0.473). MIE approaches were reported to be always used by 26.7% of endodontists and 5.3% of general dentists, whereas never using MIE was reported by 2.0% and 14.0%, respectively (Figure 1B). The corresponding numerical data are provided in Supplementary Table S1.

### 3.4. Reported Use of MIE Approaches

In the full analytical sample (*n* = 300), vital pulp therapies were reported by 75.7% of participants, minimally invasive root canal preparation by 68.0%, and minimally invasive access cavities by 23.0%. Endodontists more frequently reported the use of vital pulp therapies (90.7% vs. 60.7%), minimally invasive root canal preparation (80.0% vs. 56.0%), and minimally invasive access cavities (28.0% vs. 18.0%) than general dentists. The corresponding option-specific comparisons were statistically significant for vital pulp therapies and minimally invasive root canal preparation (both FDR-adjusted *p* < 0.001), as well as for minimally invasive access cavity use (FDR-adjusted *p* = 0.040) (Table 3).

Univariable and multivariable logistic regression analyses of factors associated with the use of MIE approaches are presented in Table 4. For minimally invasive access cavity use, endodontists had higher odds of use than general dentists in the univariable model (OR = 1.772, 95% CI: 1.024–3.065; *p* = 0.041), but this association was no longer statistically significant in the multivariable model (OR = 1.668, 95% CI: 0.763–3.648; *p* = 0.200). In the univariable analyses, ≥16 years of professional experience (OR = 4.419, 95% CI: 1.951–10.011; *p* < 0.001), employment at a university hospital (OR = 2.354, 95% CI: 1.118–4.954; *p* = 0.024), and employment in private practice/polyclinic (OR = 2.215, 95% CI: 1.050–4.673; *p* = 0.037) were also associated with higher odds of minimally invasive access cavity use. In the multivariable model, ≥16 years of professional experience (OR = 4.412, 95% CI: 1.796–10.841; *p* = 0.001) and employment at a university hospital (OR = 2.429, 95% CI: 1.009–5.845; *p* = 0.048) remained significantly associated with use.

For vital pulp therapy use, endodontists had higher odds than general dentists in both the univariable (OR = 6.298, 95% CI: 3.320–11.948; *p* < 0.001) and multivariable (OR = 5.626, 95% CI: 2.291–13.817; *p* < 0.001) models. In the univariable analyses, employment at a university hospital (OR = 5.813, 95% CI: 2.726–12.394; *p* < 0.001), employment in private practice/polyclinic (OR = 1.994, 95% CI: 1.082–3.676; *p* = 0.027), and performing 11–15 (OR = 2.461, 95% CI: 1.048–5.780; *p* = 0.039) or 16–20 (OR = 5.938, 95% CI: 1.674–21.065; *p* = 0.006) root canal treatments per week were associated with higher odds of use. In the multivariable model, employment at a university hospital (OR = 2.981, 95% CI: 1.230–7.225; *p* = 0.016) and employment in private practice/polyclinic (OR = 2.354, 95% CI: 1.187–4.667; *p* = 0.014) also remained significantly associated with vital pulp therapy use.

For minimally invasive root canal preparation, endodontists had higher odds of use than general dentists in both the univariable (OR = 3.143, 95% CI: 1.880–5.254; *p* < 0.001) and multivariable (OR = 2.238, 95% CI: 1.082–4.628; *p* = 0.030) models. In the univariable analyses, 6–10 years (OR = 2.190, 95% CI: 1.223–3.920; *p* = 0.008) and 11–15 years (OR = 3.697, 95% CI: 1.344–10.172; *p* = 0.011) of professional experience, employment at a university hospital (OR = 2.059, 95% CI: 1.128–3.758; *p* = 0.019), employment in private practice/polyclinic (OR = 2.086, 95% CI: 1.144–3.804; *p* = 0.017), and performing 11–15 (OR = 2.182, 95% CI: 1.042–4.568; *p* = 0.039) or ≥21 (OR = 4.582, 95% CI: 1.606–13.075; *p* = 0.004) root canal treatments per week were associated with higher odds of use. In the multivariable model, employment in private practice/polyclinic remained significantly associated with minimally invasive root canal preparation (OR = 3.069, 95% CI: 1.552–6.069; *p* = 0.001), whereas the professional experience and weekly treatment-volume categories were no longer statistically significant.

### 3.5. Difficulties Encountered During MIE Procedures

Univariable and multivariable logistic regression analyses of factors associated with reporting difficulties during MIE procedures are presented in Supplementary Table S2, whereas comparisons of the procedural stages at which difficulties were reported are presented in Supplementary Table S3.

Among participants using minimally invasive access cavities, none of the examined explanatory variables was significantly associated with reporting difficulty in either the univariable or multivariable models (all *p* > 0.05). The adjusted estimates from this model were relatively imprecise, as reflected by the wide 95% CIs, and should therefore be interpreted cautiously. Among participants reporting at least one difficulty, the most frequently reported stages were gaining access to the root canals (71.4%) and root canal obturation (58.9%). None of the individual procedural stages differed significantly between general dentists and endodontists after FDR adjustment (all adjusted *p* = 1.000).

For difficulties during vital pulp therapies, general dentists had higher odds of reporting difficulty than endodontists in both the univariable (OR = 6.551, 95% CI: 3.521–12.191; *p* < 0.001) and multivariable (OR = 10.420, 95% CI: 3.993–27.195; *p* < 0.001) models. Participants with 0–5 years of professional experience had higher odds of reporting difficulty than those with ≥16 years of experience in both the univariable (OR = 4.375, 95% CI: 1.654–11.575; *p* = 0.003) and multivariable (OR = 4.349, 95% CI: 1.330–14.219; *p* = 0.015) models. In the multivariable model, employment at a public hospital/oral and dental health center (OR = 2.756, 95% CI: 1.072–7.085; *p* = 0.035) and employment at a university hospital (OR = 3.000, 95% CI: 1.270–7.083; *p* = 0.012), compared with private practice/polyclinic, were also associated with higher odds of reporting difficulty. Performing 0–5 root canal treatments per week was associated with higher odds of difficulty than performing ≥ 21 treatments per week in the univariable model (OR = 3.132, 95% CI: 1.199–8.181; *p* = 0.020), but this association was not statistically significant after multivariable adjustment (*p* = 0.645). Among participants reporting difficulty during vital pulp therapies, bleeding control was more frequently reported as difficult by general dentists than by endodontists (54.8% vs. 26.9%; FDR-adjusted *p* = 0.010), whereas completion of the final restoration was more frequently reported as difficult by endodontists (42.3% vs. 15.1%; FDR-adjusted *p* = 0.005). The remaining procedural stages did not differ significantly between the professional groups after FDR adjustment.

For minimally invasive root canal preparation, general dentists had higher odds of reporting difficulty than endodontists in both the univariable (OR = 3.317, 95% CI: 1.763–6.244; *p* < 0.001) and multivariable (OR = 2.379, 95% CI: 1.045–5.417; *p* = 0.039) models. None of the professional experience categories was significantly associated with reporting difficulty in either model. In the multivariable model, employment at a public hospital/oral and dental health center was associated with higher odds of reporting difficulty than employment in private practice/polyclinic (OR = 2.651, 95% CI: 1.044–6.737; *p* = 0.040). Participants performing 0–5 or 6–10 root canal treatments per week had higher odds of reporting difficulty than those performing ≥ 21 treatments per week in the univariable analyses (OR = 3.556, 95% CI: 1.308–9.667; *p* = 0.013 and OR = 3.088, 95% CI: 1.228–7.762; *p* = 0.017, respectively). After multivariable adjustment, the association for 6–10 treatments per week remained nominally significant (OR = 3.082, 95% CI: 1.032–9.207; *p* = 0.044), whereas the association for 0–5 treatments per week was no longer statistically significant (*p* = 0.072). Given the multiplicity of analyses and the absence of a study-wide multiplicity adjustment for the regression models, the multivariable findings with nominal *p* values close to 0.05 should be regarded as exploratory and interpreted cautiously in conjunction with their effect estimates and 95% CIs. Among participants reporting difficulty during minimally invasive root canal preparation, root canal irrigation was the most frequently reported stage (66.7%). Root canal obturation was more frequently reported as difficult by general dentists than by endodontists (60.6% vs. 30.2%; FDR-adjusted *p* < 0.001), whereas root canal irrigation and irrigant activation did not differ significantly between the professional groups after FDR adjustment.

### 3.6. Reasons for MIE Use, Diagnostic Methods, and Technical Adjuncts

In the full-sample option-specific analyses, reduced loss of tooth structure was the most frequently reported reason for preferring MIE (86.3% of all participants). Endodontists more frequently selected faster healing time (33.3% vs. 19.5%; FDR-adjusted *p* = 0.019), less loss of tooth structure (94.0% vs. 78.7%; FDR-adjusted *p* < 0.001), and fewer complications (46.7% vs. 32.0%; FDR-adjusted *p* = 0.019) than general dentists. No statistically significant between-group differences were observed for less postoperative pain (26.7% vs. 18.0%; FDR-adjusted *p* = 0.098) or better patient cooperation (30.0% vs. 25.3%; FDR-adjusted *p* = 0.422).

In the corresponding full-sample option-specific analyses of reasons for not preferring MIE, inadequate training or lack of knowledge (7.3% vs. 0.7%; FDR-adjusted *p* = 0.019), difficulty of application or technical sensitivity (8.7% vs. 1.3%; FDR-adjusted *p* = 0.019), and insufficient time (8.0% vs. 1.3%; FDR-adjusted *p* = 0.026) were more frequently selected by general dentists than by endodontists. Lack of necessary equipment did not remain statistically significant after FDR adjustment (6.7% vs. 1.3%; FDR-adjusted *p* = 0.065), and no significant between-group difference was observed for the high cost of materials (4.0% vs. 0.7%; FDR-adjusted *p* = 0.151).

Endodontists more frequently reported using periapical radiography (98.7% vs. 78.0%; FDR-adjusted *p* < 0.001) and cone-beam computed tomography (CBCT) (23.3% vs. 8.0%; FDR-adjusted *p* < 0.001) than general dentists. Panoramic radiography use was similar between the professional groups (86.7% vs. 86.0%; FDR-adjusted *p* = 1.000).

Among the 122 participants who reported using magnification systems, 95.9% reported using dental loupes and 27.9% reported using a dental operating microscope. Neither dental loupe use (97.9% vs. 88.5%; FDR-adjusted *p* = 0.096) nor dental operating microscope use (29.2% vs. 23.1%; FDR-adjusted *p* = 0.764) differed significantly between endodontists and general dentists. Detailed results regarding reasons for MIE use and non-use, radiographic diagnostic methods, and types of magnification systems are presented in Supplementary Table S4.

Regression results for the use of magnification systems and specialized endodontic burs for access cavity preparation or refinement are presented in Supplementary Table S5. Endodontists had higher odds of using magnification systems than general dentists in both the univariable (OR = 8.479, 95% CI: 4.949–14.526; *p* < 0.001) and multivariable (OR = 9.131, 95% CI: 4.057–20.554; *p* < 0.001) models. In the univariable analyses, participants with 6–10 years (OR = 2.744, 95% CI: 1.590–4.737; *p* < 0.001), 11–15 years (OR = 2.969, 95% CI: 1.368–6.444; *p* = 0.006), and ≥16 years (OR = 2.484, 95% CI: 1.135–5.435; *p* = 0.023) of professional experience had higher odds of magnification system use than those with 0–5 years of experience. Employment at a university hospital (OR = 19.634, 95% CI: 8.246–46.749; *p* < 0.001) or in private practice/polyclinic (OR = 9.298, 95% CI: 3.929–22.002; *p* < 0.001), compared with employment at a public hospital/oral and dental health center, was also associated with higher odds of magnification system use in the univariable analyses. Participants performing 11–15 root canal treatments per week had higher odds of magnification system use than those performing 0–5 treatments per week (OR = 2.229, 95% CI: 1.104–4.497; *p* = 0.025).

In the multivariable model, 6–10 years (OR = 2.173, 95% CI: 1.025–4.608; *p* = 0.043) and ≥16 years (OR = 2.893, 95% CI: 1.035–8.087; *p* = 0.043) of professional experience remained associated with higher odds of magnification system use compared with 0–5 years. Employment at a university hospital (OR = 15.975, 95% CI: 5.509–46.325; *p* < 0.001) and in private practice/polyclinic (OR = 18.610, 95% CI: 6.333–54.683; *p* < 0.001) also remained significantly associated with higher odds of magnification system use. None of the weekly root canal treatment categories was significantly associated with magnification system use in the multivariable model.

Endodontists also had higher odds of using specialized endodontic burs for access cavity preparation or refinement than general dentists in both the univariable (OR = 9.070, 95% CI: 5.369–15.324; *p* < 0.001) and multivariable (OR = 5.970, 95% CI: 2.794–12.755; *p* < 0.001) models. In the univariable analyses, participants with 6–10 years (OR = 2.356, 95% CI: 1.387–4.003; *p* = 0.002) and ≥16 years (OR = 2.750, 95% CI: 1.255–6.024; *p* = 0.011) of professional experience had higher odds of specialized bur use than those with 0–5 years of experience. Employment at a university hospital (OR = 8.105, 95% CI: 4.224–15.550; *p* < 0.001) or in private practice/polyclinic (OR = 2.725, 95% CI: 1.454–5.108; *p* = 0.002), and performing 16–20 (OR = 2.786, 95% CI: 1.266–6.131; *p* = 0.011) or ≥21 (OR = 3.450, 95% CI: 1.450–8.208; *p* = 0.005) root canal treatments per week were also associated with higher odds of specialized bur use in the univariable analyses.

In the multivariable model, ≥16 years of professional experience remained associated with higher odds of specialized bur use compared with 0–5 years (OR = 4.229, 95% CI: 1.541–11.600; *p* = 0.005). Employment at a university hospital (OR = 10.282, 95% CI: 4.081–25.903; *p* < 0.001) and in private practice/polyclinic (OR = 5.928, 95% CI: 2.447–14.363; *p* < 0.001) also remained significant, as did performing ≥ 21 root canal treatments per week compared with 0–5 treatments per week (OR = 3.701, 95% CI: 1.083–12.644; *p* = 0.037).

### 3.7. Perceptions of MIE, Perceived Barriers, and Recommendations

Overall, 50.3% of participants believed that performing root canal treatment using MIE approaches increased treatment success, 12.3% did not, and 37.3% were unsure. The distribution of responses did not differ significantly between general dentists and endodontists after FDR adjustment (FDR-adjusted *p* = 0.541).

The most frequently reported barriers to the routine clinical implementation of MIE were inadequate training or lack of knowledge (68.0%), difficulty changing established habits (57.7%), equipment cost (54.0%), and technical difficulty or a long learning curve (53.3%). None of the option-specific comparisons differed significantly between general dentists and endodontists after FDR adjustment (all FDR-adjusted *p* > 0.05).

The most frequently recommended measures for promoting wider adoption of MIE in Türkiye were greater emphasis on MIE in undergraduate or specialty education (85.3%), provision of equipment support to clinics (76.3%), and increased continuing education programs (63.3%). Although provision of equipment support to clinics was more frequently selected by endodontists than by general dentists in the unadjusted comparison (82.0% vs. 70.7%; *p* = 0.021), this difference was no longer statistically significant after FDR adjustment (FDR-adjusted *p* = 0.231). None of the remaining recommendation options differed significantly between the professional groups after FDR adjustment. Detailed results are presented in Supplementary Table S6.

## 4. Discussion

This study compared general dentists and endodontists in Türkiye in terms of their self-perceived knowledge of MIE, self-reported clinical practices, and perceived difficulties. While 68.7% of endodontists reported having an adequate level of knowledge of MIE, the corresponding proportion among general dentists was 16.7%. The proportion of participants who reported always or usually using MIE was also higher among endodontists than among general dentists (68.0% vs. 43.3%). Endodontists more frequently reported the use of minimally invasive access cavities, vital pulp therapies, minimally invasive root canal preparation, magnification systems, and specialized endodontic burs. In multivariable models including professional group, professional experience, institutional setting, and weekly root canal treatment volume, professional group remained significantly associated with most of the binary outcomes examined, although the association with minimally invasive access cavity use was no longer statistically significant. Nevertheless, given the cross-sectional and self-reported nature of the study, these findings should be interpreted as associations rather than causal relationships.

A previous study conducted in Türkiye reported that 63.5% of dentists were aware of MIE, whereas only 31.2% reported routinely applying this approach [23]. The relatively limited number of endodontists in the sample of that study made detailed comparisons between professional groups difficult. In the present study, the equal numbers of participants in the two professional groups provided a balanced sample for between-group comparisons. The findings regarding education also point to a potential contributor to this difference: only 15.7% of participants considered the MIE education they had received to be adequate, whereas 51.0% considered it partially adequate and 28.7% considered it inadequate. General dentists more frequently perceived their education as inadequate, whereas endodontists more frequently reported congresses, courses, and academic publications as sources of information; general dentists, in contrast, more frequently reported social media and the experiences of colleagues. A study conducted in Norway reported that undergraduate education may have a long-term role in the adoption of endodontic technological adjuncts and their translation into clinical practice, while continuing professional education was also an important source of learning among clinicians who had graduated longer ago [28]. A study involving undergraduate dental students and postgraduate endodontic students from 10 countries found that preferences for the management of deep caries and pulp exposure varied according to the participants’ level of training and the clinical scenario [29]. In a study conducted in Syria, 62.3% of participants reported having received some education in minimally invasive dentistry, whereas only 25.0% reported having received comprehensive training [30]. Although these studies did not assess exactly the same clinical domain, collectively they suggest that knowledge transfer should be supported by case selection and practical skills training.

The proportion of participants who reported using minimally invasive access cavities was 18.0% among general dentists and 28.0% among endodontists. Although minimally invasive access cavity use was more frequently reported by endodontists in the univariable comparison, the association between professional group and use of this approach was no longer statistically significant in the multivariable model (OR = 1.668, 95% CI: 0.763–3.648; *p* = 0.200). In a survey of members of the AAE, 57% of participants preferred traditional access cavities and 43% preferred conservative access cavities, whereas ultraconservative designs were reported only rarely [31]. In the previous study from Türkiye, traditional access cavities and conservative designs were reported by 56.8% and 42.3% of participants, respectively [23]. However, because the question formats, samples, and definitions differed across studies, these proportions should not be directly compared. Although conservative access designs may reduce dentin removal, they have not demonstrated a consistent advantage in terms of fracture resistance or other treatment-related outcomes, and restricted access cavities have been reported to potentially complicate canal orifice location, cleaning and shaping of the root canal system, and obturation [17,19,32]. Indeed, 56 of the 69 participants who reported using minimally invasive access cavities reported at least one difficulty, with gaining access to the root canals and root canal obturation being the most frequently reported stages. These findings are consistent with the literature suggesting that conservative access designs should not be regarded as a uniform treatment goal, but rather should be considered on a case-specific basis by balancing anatomy, visualization, disinfection requirements, and technical feasibility [17,19,32].

One of the most pronounced differences observed in the present study concerned the use of vital pulp therapies. The proportion of participants who reported performing vital pulp therapies was 60.7% among general dentists and 90.7% among endodontists. In the multivariable analysis, endodontists had substantially higher odds of reporting the use of vital pulp therapies than general dentists (OR = 5.626, 95% CI: 2.291–13.817; *p* < 0.001). The magnitude of this association should not be interpreted as reflecting professional group alone. Differences in referral patterns, case mix, exposure to cases suitable for vital pulp therapy, practice setting, access to specialized resources, and educational exposure may also influence the likelihood of performing these procedures. Although the multivariable model adjusted for professional experience, institutional setting, and weekly root canal treatment volume in addition to professional group, referral patterns, case mix, exposure to suitable cases, access to specialized resources, and educational exposure were not directly measured. Residual confounding by these and other unmeasured professional or practice-related characteristics therefore cannot be excluded. A survey involving members of the Irish and Italian endodontic societies showed that preferences for the management of pulp exposure varied according to country, training, and clinical scenario [33]. A recent study from Spain similarly demonstrated that some preferences regarding the management of deep caries and pulp exposure were not fully consistent with current vital pulp therapy recommendations [34]. Because education, healthcare delivery, and clinical scenarios differ across countries, direct ranking of reported utilization rates is not appropriate. Nevertheless, the ESE recommendations support preservation of pulp vitality and the use of vital pulp therapy in appropriately selected cases, provided that accurate diagnosis and appropriate case selection are ensured [1,15].

Among participants who performed vital pulp therapies, general dentists had substantially higher odds of reporting at least one difficulty than endodontists in the multivariable model (OR = 10.420, 95% CI: 3.993–27.195; *p* < 0.001). Isolation, bleeding control, application of the pulp-protective agent, and completion of the final restoration were among the prominent stages at which difficulties were reported. After FDR adjustment, bleeding control was more frequently reported as difficult by general dentists, whereas completion of the final restoration was more frequently reported as difficult by endodontists. It should be noted that the percentages for individual procedural stages were calculated only among participants who reported at least one difficulty. In a study from the United Kingdom, insufficient knowledge and clinical confidence, access to materials and equipment, time constraints, and issues related to funding and remuneration were reported as major barriers to the use of vital pulp therapy [35]. Our findings suggest that vital pulp therapy education may benefit from addressing not only indications and case selection but also specific clinical stages such as isolation, bleeding control, material application, and coronal restoration.

The proportion of participants who reported using minimally invasive root canal preparation was 56.0% among general dentists and 80.0% among endodontists. Professional group remained significantly associated with the use of minimally invasive root canal preparation in the multivariable model, with endodontists having higher odds of reporting its use than general dentists (OR = 2.238, 95% CI: 1.082–4.628; *p* = 0.030). General dentists also had higher odds of reporting at least one difficulty during minimally invasive root canal preparation than endodontists after multivariable adjustment (OR = 2.379, 95% CI: 1.045–5.417; *p* = 0.039). Irrigation and root canal obturation were among the most frequently reported stages at which difficulties were encountered. After FDR adjustment, root canal obturation was more frequently reported as difficult by general dentists than by endodontists. A computational fluid dynamics study conducted in minimally prepared root canals demonstrated that irrigant flow and apical pressure patterns were influenced by apical preparation size, canal taper, needle design, and flow rate [36]. Therefore, the objective of dentin preservation should not be considered independently of the need to achieve safe and effective irrigation.

The use of magnification systems was reported by 17.3% of general dentists and 64.0% of endodontists, whereas the use of specialized endodontic burs for access cavity preparation or refinement was reported by 22.7% and 72.7%, respectively. In the adjusted models, professional group remained associated with both magnification system use and the use of specialized endodontic burs. The multivariable analyses also indicated that the reported use of these technical adjuncts was associated not only with professional group but also with characteristics of professional experience, institutional setting, and, for specialized bur use, weekly treatment volume. These self-reported findings are consistent with prospectively recorded clinical treatment data. Nosrat et al. [37] prospectively evaluated 1705 nonsurgical root canal treatments and reported that endodontists used adjuncts such as high magnification, rubber dam isolation, irrigation activation, and electronic apex locators more frequently than general dentists. In a study involving 1358 clinicians from Spain and Latin America, the use of advanced endodontic technologies varied according to educational level and geographic region [38]. A global survey conducted among members of endodontic societies also reported differences in technology use across countries and geographic regions [39]. A study involving endodontists and postgraduate endodontic students in Türkiye emphasized the importance of accessibility as well as economic and logistical factors in the use of magnification systems [40]. Although these studies did not assess MIE in exactly the same manner as the present study, they support the possibility that specialty training is an important component underlying differences in technology use.

The more frequent reported use of CBCT, in addition to periapical radiography, among endodontists may reflect the more frequent use of advanced imaging in complex cases. However, because the survey did not collect indication-specific clinical data, we could not assess whether CBCT use was appropriate in individual cases. The ESE does not recommend routine CBCT use, but rather its use in selected situations in which conventional imaging is insufficient and the additional information obtained may influence treatment decisions [41]. Accordingly, the between-group difference should not be interpreted directly as evidence of better or more appropriate clinical practice.

The finding that 86.3% of participants reported reduced loss of tooth structure as a reason for preferring MIE suggests broad recognition of the fundamental tissue-preservation objective of the approach. Nevertheless, only 50.3% of participants believed that performing root canal treatments using MIE approaches increased the success rate, while 37.3% were unsure. This cautious perception is consistent with the limited evidence regarding the long-term clinical superiority of minimally invasive access cavity designs over traditional access designs [17,19,32]. The finding that inadequate training and lack of knowledge were the most frequently reported barriers to the wider adoption of MIE (68.0%) suggests that structured continuing professional development programs may be beneficial. None of the individual barrier options differed significantly between general dentists and endodontists after FDR adjustment. However, in addition to individual education, access to magnification, imaging, and irrigation systems, as well as clinical workflow considerations, should also be taken into account.

Among the strengths of this study were the equal numbers of participating general dentists and endodontists, which provided balanced group sizes for between-group comparisons. In addition, MIE was evaluated not only in terms of self-perceived knowledge but also with respect to minimally invasive access cavities, vital pulp therapies, minimally invasive root canal preparation, imaging methods, magnification systems, reported procedural difficulties, and perceived barriers. For conditionally applicable analyses, such as procedural stages of difficulty and magnification system types, the relevant denominators were restricted to participants for whom those questions were applicable, allowing these findings to be interpreted within the corresponding clinical context.

Nevertheless, this study has several limitations. The use of non-probability, voluntary-response online sampling may have introduced selection and response biases, and the sample cannot be assumed to be representative of all general dentists and endodontists practicing in Türkiye. Because the distribution channels potentially overlapped and the number of unique individuals reached could not be determined, an overall response rate could not be calculated. Accordingly, the observed proportions and between-group associations should be interpreted as findings from the participating sample rather than as population-level estimates for general dentists and endodontists practicing in Türkiye. Self-reported outcomes are susceptible to recall and social desirability biases, and actual clinical practice was not verified against clinical records.

Although the content-validity panel consisted exclusively of endodontists selected for their clinical and academic expertise in MIE, inclusion of general dentists in the expert panel could have provided additional perspectives regarding the relevance, clarity, and interpretation of the questionnaire items for the general dentist population. The subsequent pilot study included both general dentists and endodontists and specifically evaluated question comprehensibility, response-option appropriateness, conditional skip logic, and overall feasibility. However, pilot testing does not fully substitute for the inclusion of general dentists in the formal content-validity panel, and this limitation should be considered when interpreting the content validity of the questionnaire across the two professional groups.

Because the questionnaire assessed several distinct domains of knowledge and clinical practice rather than constituting a single multi-item psychometric scale, no total score, factor analysis, or Cronbach’s alpha was calculated. Although the content-validity assessment and pilot testing supported the scope and comprehensibility of the questions, the absence of standardized operational definitions may have resulted in heterogeneous interpretation of the individual MIE approaches. In particular, participants may have differed in how they interpreted vital pulp therapy and minimally invasive root canal preparation. Consequently, some degree of misclassification of self-reported clinical use cannot be excluded, and the extent or direction of this potential bias cannot be determined from the present data. Self-perceived knowledge and frequency of self-reported clinical use were assessed using single-item outcomes and may therefore be susceptible to measurement error. Because the responses to multiple-response items were obtained from the same participants, the individual response options were not statistically independent; therefore, option-level comparisons for these items should be interpreted as exploratory.

From an analytical perspective, although the overall analytical sample comprised 300 participants, the effective sample size was smaller for some outcome-specific regression analyses restricted to eligible participant subgroups. This was particularly relevant to the minimally invasive access cavity difficulty model, in which relatively few participants were in the no-difficulty category relative to the complexity of the adjusted model. The corresponding adjusted estimates therefore had limited precision, as reflected by their wide 95% CIs, and should be interpreted cautiously. In addition, the Hosmer–Lemeshow test indicated evidence of lack of fit for the multivariable model examining specialized endodontic bur use; estimates from this model should therefore be interpreted with additional caution. Residual confounding by unmeasured professional, educational, or practice-related characteristics cannot be excluded despite adjustment for professional group, professional experience, institutional setting, and weekly root canal treatment volume. The ordinal nature of the self-perceived knowledge and frequency-of-use items was evaluated using cross-tabulations, and multivariable ordinal regression models were not fitted for these outcomes. Because numerous statistical comparisons were performed across several distinct questionnaire domains, the possibility of inflated Type I error should be considered. Although multiplicity adjustment was applied within relevant families of comparisons, no single study-wide multiplicity adjustment covering all secondary and exploratory analyses was applied. Consequently, nominal *p* values from analyses not subject to multiplicity adjustment, particularly those close to 0.05, should be regarded as exploratory rather than confirmatory, and interpretation should consider the magnitude and precision of the corresponding effect estimates and 95% confidence intervals rather than statistical significance alone. The presentation of specific stages of difficulty only among participants who reported at least one difficulty also means that these percentages should not be generalized to all participants who performed the corresponding procedure. Future studies would benefit from probability-based or stratified sampling, validation against clinical records, and prespecified multivariable models for the principal ordinal outcomes.

Overall, these findings suggest that MIE education should emphasize not only broader adoption of the approach but also appropriate case selection and the balance among tissue preservation, safe disinfection, and technical feasibility.

## 5. Conclusions

Within the limitations of this cross-sectional survey, endodontists reported higher self-perceived knowledge of MIE and more frequent self-reported clinical use of MIE approaches than general dentists. Professional and practice-related characteristics were associated with several MIE-related outcomes in the multivariable analyses. Inadequate training or lack of knowledge was the most frequently reported perceived barrier to the routine implementation of MIE. These findings reflect perceived educational gaps and observed associations within the participating sample rather than objectively measured deficiencies in knowledge, training, or clinical competence. They suggest the potential value of greater emphasis on practical, case-based MIE education that integrates appropriate case selection with the principles of tissue preservation, safe and effective disinfection, and technical feasibility. Given the non-probability sampling, cross-sectional design, and self-reported nature of the data, the observed associations should not be interpreted as causal. Furthermore, the findings should not be generalized as representative of all general dentists and endodontists practicing in Türkiye.

## Figures and Tables

**Table 1:** Comparison of demographic and professional characteristics between general dentists and endodontists.

Characteristic	General dentists *n* (%)	Endodontists *n* (%)	Total *n* (%)	Test Statistic	*p*	*adj. p*	ES (95% CI)
Sex				1.357	0.244 ^x^	0.244	0.067 [0.058–0.130]
Female	80 (53.3)	90 (60.0)	170 (56.7)				
Male	70 (46.7)	60 (40.0)	130 (43.3)				
Age (years)				38.005	<0.001 ^y^	<0.001	0.353 [0.249–0.468]
23–30	100 (66.7) ^a^	62 (41.3) ^b^	162 (54.0)				
31–40	27 (18.0) ^a^	77 (51.3) ^b^	104 (34.7)				
41–50	12 (8.0)	5 (3.3)	17 (5.7)				
≥51	11 (7.3)	6 (4.0)	17 (5.7)				
Professional experience in dentistry				52.763	<0.001 ^y^	<0.001	0.416 [0.309–0.531]
0–5 years	91 (60.7) ^a^	40 (26.7) ^b^	131 (43.7)				
6–10 years	28 (18.7) ^a^	74 (49.3) ^b^	102 (34.0)				
11–15 years	9 (6.0) ^a^	25 (16.7) ^b^	34 (11.3)				
≥16 years	22 (14.7) ^a^	11 (7.3) ^b^	33 (11.0)				
Institution of employment				52.837	<0.001 ^x^	<0.001	0.420 [0.313–0.536]
Public hospital/oral and dental health center	55 (36.7) ^a^	32 (21.3) ^b^	87 (29.0)				
University hospital	23 (15.3) ^a^	83 (55.3) ^b^	106 (35.3)				
Private practice/polyclinic	72 (48.0) ^a^	35 (23.3) ^b^	107 (35.7)				
Weekly number of root canal treatments				65.959	<0.001 ^y^	<0.001	0.457 [0.351–0.572]
0–5	59 (39.3) ^a^	21 (14.0) ^b^	80 (26.7)				
6–10	59 (39.3) ^a^	33 (22.0) ^b^	92 (30.7)				
11–15	19 (12.7) ^a^	36 (24.0) ^b^	55 (18.3)				
16–20	10 (6.7) ^a^	30 (20.0) ^b^	40 (13.3)				
≥21	3 (2.0) ^a^	30 (20.0) ^b^	33 (11.0)				

^x^ Pearson’s chi-square test; ^y^ Fisher’s exact test with Monte Carlo simulation. Superscript letters a and b indicate the results of Bonferroni-adjusted Z tests for pairwise comparisons of column proportions; values within the same row that share the same superscript letter do not differ significantly, whereas values with different superscript letters indicate a statistically significant difference between groups. Benjamini–Hochberg false discovery rate (FDR) adjustment was applied to the comparisons reported in the table; adjusted *p* values are presented as *adj. p.* ES, effect size; CI, confidence interval. Phi was used as the effect-size measure for 2 × 2 contingency tables, whereas Cramér’s V was used for larger contingency tables. The 95% confidence intervals for the effect sizes were calculated using the percentile bootstrap method with 2000 resamples. Values are presented as *n* (%).

**Table 2:** Comparison of the perceived adequacy of minimally invasive endodontics (MIE) training and preferred information sources between general dentists and endodontists.

Characteristic	General Dentists *n* (%)	Endodontists *n* (%)	Total *n* (%)	Test Statistic	*p*	*adj. p*	ES (95% CI)
Perceived adequacy of training on MIE approaches				14.939	0.002 ^y^	0.002	0.223 [0.132–0.339]
Adequate	17 (11.3) ^a^	30 (20.0) ^b^	47 (15.7)				
Partially adequate	68 (45.3) ^a^	85 (56.7) ^b^	153 (51.0)				
Inadequate	57 (38.0) ^a^	29 (19.3) ^b^	86 (28.7)				
No training received	8 (5.3)	6 (4.0)	14 (4.7)				
Preferred sources of information about MIE approaches *							
Congresses/seminars/courses	76 (50.7)	118 (78.7)	194 (64.7)	25.734	<0.001 ^x^	<0.001	0.293 [0.175–0.391]
Academic articles/publications	61 (40.7)	126 (84.0)	187 (62.3)	59.983	<0.001 ^x^	<0.001	0.447 [0.349–0.544]
Online education	78 (52.0)	62 (41.3)	140 (46.7)	3.429	0.064 ^x^	0.064	0.107 [0.010–0.223]
Social media platforms	103 (68.7)	68 (45.3)	171 (57.0)	16.660	<0.001 ^x^	<0.001	0.236 [0.130–0.347]
Experience sharing with other dentists	93 (62.0)	52 (34.7)	145 (48.3)	22.438	<0.001 ^x^	<0.001	0.274 [0.166–0.386]

* Multiple-response item; each response option was coded as a separate binary variable and analyzed separately between professional groups using an option-specific 2 × 2 comparison. Superscript x indicates Pearson’s chi-square test, and superscript y indicates Fisher’s exact test with Monte Carlo simulation. For the single-response item, superscript letters a and b indicate the results of Bonferroni-adjusted Z tests for pairwise comparisons of column proportions; values within the same row that share the same superscript letter do not differ significantly, whereas values with different superscript letters differ significantly. Benjamini–Hochberg false discovery rate (FDR) adjustment was applied to the reported comparisons; adjusted *p* values are presented as *adj. p*. ES, effect size; CI, confidence interval. Phi was used for 2 × 2 comparisons and Cramér’s V for larger contingency tables. The 95% confidence intervals for the effect sizes were calculated using 2000 bootstrap resamples. Values are presented as *n* (%).

**Table 3:** Comparison of minimally invasive endodontic (MIE) approaches used by general dentists and endodontists.

	General Dentists (*n* = 150), *n* (%)	Endodontists (*n* = 150), *n* (%)	Total (*n* = 300), *n* (%)	Test Statistic	*p*	*adj. p*	ES (95% CI)
Which MIE approaches do you use in clinical practice? *							
Minimally invasive access cavity	27 (18.0)	42 (28.0)	69 (23.0)	4.235	0.040 ^x^	0.040	0.119 [0.017–0.228]
Vital pulp therapies	91 (60.7)	136 (90.7)	227 (75.7)	36.660	<0.001 ^x^	<0.001	0.350 [0.247–0.444]
Minimally invasive root canal preparation	84 (56.0)	120 (80.0)	204 (68.0)	19.853	<0.001 ^x^	<0.001	0.257 [0.140–0.360]

* Multiple-response item; each response option was coded as a separate binary variable (1 = selected, 0 = not selected) and analyzed separately between professional groups using an option-specific 2 × 2 contingency table. ^x^ Pearson’s chi-square test. The Benjamini–Hochberg false discovery rate (FDR) procedure was applied across the three option-specific comparisons; adjusted *p* values are presented as *adj. p*. ES, effect size; CI, confidence interval. Phi was used as the effect-size measure for the 2 × 2 comparisons. The 95% confidence intervals for Phi were calculated using the percentile bootstrap method with 2000 resamples. Values are presented as frequency (percentage). MIE, minimally invasive endodontics.

**Table 4:** Univariable and multivariable logistic regression analyses of factors associated with the use of minimally invasive endodontic approaches.

Variable	Not Used, *n* (%)	Used, *n* (%)	Univariable Model OR (95% CI)	*p*	Multivariable Model OR (95% CI)	*p*
Use of minimally invasive access cavity						
Professional group						
General dentists	123 (82.0)	27 (18.0)	Reference	—	Reference	—
Endodontists	108 (72.0)	42 (28.0)	1.772 (1.024–3.065)	0.041	1.668 (0.763–3.648)	0.200
Professional experience in dentistry						
0–5 years	108 (82.4)	23 (17.6)	Reference	—	Reference	—
6–10 years	81 (79.4)	21 (20.6)	1.217 (0.630–2.351)	0.558	1.028 (0.492–2.148)	0.941
11–15 years	25 (73.5)	9 (26.5)	1.690 (0.698–4.096)	0.245	1.293 (0.495–3.376)	0.599
≥16 years	17 (51.5)	16 (48.5)	4.419 (1.951–10.011)	<0.001	4.412 (1.796–10.841)	0.001
Institution of employment						
Public hospital/oral and dental health center	75 (86.2)	12 (13.8)	Reference	—	Reference	—
University hospital	77 (72.6)	29 (27.4)	2.354 (1.118–4.954)	0.024	2.429 (1.009–5.845)	0.048
Private practice/polyclinic	79 (73.8)	28 (26.2)	2.215 (1.050–4.673)	0.037	2.218 (0.992–4.962)	0.052
Weekly number of root canal treatments						
0–5	59 (73.8)	21 (26.3)	Reference	—	Reference	—
6–10	75 (81.5)	17 (18.5)	0.637 (0.309–1.315)	0.222	0.851 (0.380–1.906)	0.695
11–15	44 (80.0)	11 (20.0)	0.702 (0.307–1.607)	0.403	0.688 (0.274–1.729)	0.426
16–20	29 (72.5)	11 (27.5)	1.066 (0.454–2.504)	0.884	1.185 (0.448–3.136)	0.733
≥21	24 (72.7)	9 (27.3)	1.054 (0.423–2.627)	0.911	1.456 (0.479–4.424)	0.508
Use of vital pulp therapies						
Professional group						
General dentists	59 (39.3)	91 (60.7)	Reference	—	Reference	—
Endodontists	14 (9.3)	136 (90.7)	6.298 (3.320–11.948)	<0.001	5.626 (2.291–13.817)	<0.001
Professional experience in dentistry						
0–5 years	33 (25.2)	98 (74.8)	Reference	—	Reference	—
6–10 years	22 (21.6)	80 (78.4)	1.224 (0.662–2.265)	0.519	0.592 (0.276–1.269)	0.178
11–15 years	7 (20.6)	27 (79.4)	1.299 (0.518–3.260)	0.578	0.600 (0.200–1.803)	0.363
≥16 years	11 (33.3)	22 (66.6)	0.673 (0.295–1.536)	0.347	0.607 (0.235–1.562)	0.300
Institution of employment						
Public hospital/oral and dental health center	35 (40.2)	52 (59.8)	Reference	—	Reference	—
University hospital	11 (10.4)	95 (89.6)	5.813 (2.726–12.394)	<0.001	2.981 (1.230–7.225)	0.016
Private practice/polyclinic	27 (25.2)	80 (74.8)	1.994 (1.082–3.676)	0.027	2.354 (1.187–4.667)	0.014
Weekly number of root canal treatments						
0–5	26 (32.5)	54 (67.5)	Reference	—	Reference	—
6–10	29 (31.5)	63 (68.5)	1.046 (0.550–1.988)	0.891	1.004 (0.475–2.121)	0.992
11–15	9 (16.4)	46 (83.6)	2.461 (1.048–5.780)	0.039	1.180 (0.449–3.099)	0.737
16–20	3 (7.5)	37 (92.5)	5.938 (1.674–21.065)	0.006	3.112 (0.792–12.227)	0.104
≥21	6 (18.2)	27 (81.8)	2.167 (0.797–5.894)	0.130	1.028 (0.293–3.608)	0.966
Use of minimally invasive root canal preparation						
Professional group						
General dentists	66 (44.0)	84 (56.0)	Reference	—	Reference	—
Endodontists	30 (20.0)	120 (80.0)	3.143 (1.880–5.254)	<0.001	2.238 (1.082–4.628)	0.030
Professional experience in dentistry						
0–5 years	51 (38.9)	80 (61.1)	Reference	—	Reference	—
6–10 years	23 (22.5)	79 (77.5)	2.190 (1.223–3.920)	0.008	1.608 (0.825–3.134)	0.163
11–15 years	5 (14.7)	29 (85.3)	3.697 (1.344–10.172)	0.011	2.702 (0.916–7.973)	0.072
≥16 years	17 (51.5)	16 (48.5)	0.600 (0.278–1.293)	0.192	0.521 (0.222–1.221)	0.134
Institution of employment						
Public hospital/oral and dental health center	38 (43.7)	49 (56.3)	Reference	—	Reference	—
University hospital	29 (27.4)	77 (72.6)	2.059 (1.128–3.758)	0.019	1.644 (0.772–3.500)	0.198
Private practice/polyclinic	29 (27.1)	78 (72.9)	2.086 (1.144–3.804)	0.017	3.069 (1.552–6.069)	0.001
Weekly number of root canal treatments						
0–5	36 (45.0)	44 (55.0)	Reference	—	Reference	—
6–10	29 (31.5)	63 (68.5)	1.777 (0.954–3.312)	0.070	1.314 (0.658–2.624)	0.439
11–15	15 (27.3)	40 (72.7)	2.182 (1.042–4.568)	0.039	1.302 (0.572–2.963)	0.530
16–20	11 (27.5)	29 (72.5)	2.157 (0.948–4.907)	0.067	1.279 (0.508–3.221)	0.602
≥21	5 (15.2)	28 (84.8)	4.582 (1.606–13.075)	0.004	2.767 (0.822–9.307)	0.100

OR, odds ratio; CI, confidence interval. Values are presented as *n* (%). Reference categories are indicated by “Reference.” Multivariable models included professional group, professional experience in dentistry, institution of employment, and weekly number of root canal treatments simultaneously. Model fit: minimally invasive access cavity, Cox & Snell R^2^ = 0.079, Nagelkerke R^2^ = 0.120, Hosmer–Lemeshow χ^2^ = 3.293, *p* = 0.915; vital pulp therapies, Cox & Snell R^2^ = 0.171, Nagelkerke R^2^ = 0.255, Hosmer–Lemeshow χ^2^ = 7.527, *p* = 0.481; minimally invasive root canal preparation, Cox & Snell R^2^ = 0.129, Nagelkerke R^2^ = 0.181, Hosmer–Lemeshow χ^2^ = 3.348, *p* = 0.911.

## Data Availability

The data presented in this study are available from the corresponding author upon reasonable request. The data are not publicly available due to ethical and privacy considerations related to participant-level survey data.

## Supplementary Materials

The following supporting information can be downloaded at: https://www.mdpi.com/article/10.3390/healthcare14193279/s1. Supplementary File S1: Completed STROBE Checklist for Cross-Sectional Studies; Supplementary File S2: English Translation of the Questionnaire Administered to Participants; Supplementary Table S1: Comparison of self-perceived knowledge levels regarding MIE and the frequency of self-reported MIE use in clinical practice between general dentists and endodontists; Supplementary Table S2: Univariable and multivariable logistic regression analyses of factors associated with reporting difficulties during minimally invasive endodontic procedures; Supplementary Table S3: Comparison of procedural stages at which difficulties were reported during minimally invasive endodontic procedures between general dentists and endodontists; Supplementary Table S4: Comparison of reported reasons for preferring and not preferring MIE approaches, radiographic diagnostic methods, and magnification systems used between general dentists and endodontists; Supplementary Table S5: Univariable and multivariable logistic regression analyses of factors associated with the use of magnification systems and specialized endodontic burs for access cavity preparation or refinement; Supplementary Table S6: Perceptions regarding MIE, perceived barriers to routine clinical implementation, and recommendations for wider adoption among general dentists and endodontists.

## Abbreviations

The following abbreviations are used in this manuscript:

MIE	Minimally invasive endodontics
CBCT	Cone-beam computed tomography
I-CVI	Item-level content validity index
S-CVI/Ave	Scale-level content validity index/average
OR	Odds ratio
CI	Confidence interval
FDR	False discovery rate
ESE	European Society of Endodontology
AAE	American Association of Endodontists
STROBE	Strengthening the Reporting of Observational Studies in Epidemiology
