# Supplementary material for: Self-Perceived Knowledge and Self-Reported Clinical Use of Minimally Invasive Endodontics Among General Dentists and Endodontists in Türkiye: A Cross-Sectional Survey
Source: Healthcare (Basel). 2026 Oct 2;14(19):3279. doi: 10.3390/healthcare14193279 (PMC13665129; doi:10.3390/healthcare14193279)
Supplement: Supplementary file 1 [file healthcare-14-03279-s001.zip › Supplementary File S1.pdf]

Supplementary File S1. Completed Strengthening the Reporting of Observational Studies in Epidemiology (STROBE) Checklist for Cross-Sectional Studies

Checklist item	No.	Reporting item	Where reported
**TITLE AND ABSTRACT**			
**Title and abstract**	1a	Identify the cross-sectional study design in the title or abstract.	Title p. 1; Abstract pp. 1-2
**Title and abstract**	1b	Provide an informative and balanced summary of methods and findings.	Abstract pp. 1-2
**INTRODUCTION**			
**Background / rationale**	2	Explain the scientific background and rationale for the study.	Introduction pp. 2-3
**Objectives**	3	State the specific study objectives and any prespecified hypotheses, if applicable.	Introduction p. 3, final paragraph
**METHODS**			
**Study design**	4	Present the key elements of the study design early in the manuscript.	Section 2.1, p. 3
**Setting**	5	Describe the setting, location, and relevant dates, including the data-collection period.	Sections 2.1 and 2.4, pp. 3 and 5-6
**Participants**	6a	Report eligibility criteria and the sources and methods used to recruit/select participants.	Section 2.4, p. 5; Supplementary File S2
**Variables**	7	Define the outcomes, explanatory variables, potential confounders, and other variables of interest.	Sections 2.5-2.6, pp. 6-7; Supplementary File S2
**Data sources / measurement**	8*	Describe data sources and measurement/assessment methods and their comparability between groups.	Sections 2.2 and 2.5, pp. 4 and 6; Supplementary File S2
**Bias**	9	Describe efforts to address potential sources of bias.	Sections 2.2–2.4, pp. 4-6; Section 2.6, pp. 6–7**;** Discussion limitations, pp. 20-21
**Study size**	10	Explain how the required study size was determined.	Section 2.3, p. 5
**Quantitative variables**	11	Explain how quantitative variables were handled and, where applicable, how categories were defined.	Section 2.6, pp. 6-7; Supplementary File S2; Table 1, p. 8. Age, professional experience, and weekly number of root canal treatments were collected and analyzed using prespecified categorical response ranges; no continuous quantitative variables were analyzed.
**Statistical methods**	12a	Describe statistical methods, including methods used to address confounding.	Section 2.6, pp. 6-7
**Statistical methods**	12b	Describe subgroup or interaction analyses, if performed.	No formal subgroup or interaction analyses were performed.
**Statistical methods**	12c	Explain how missing data and structurally inapplicable responses were handled.	Sections 2.4 and 2.6, pp. 5-7: eight incomplete responses were excluded; structurally inapplicable responses were handled according to the survey skip logic using analysis-specific denominators or explicit 0 coding for specified option-level analyses.
**Statistical methods**	12d	Describe analytical methods that account for the sampling strategy, if applicable.	Section 2.4, pp. 5-6: non-probability voluntary sampling was used; no individual-level random sampling was performed
**Statistical methods**	12e	Describe sensitivity analyses, if performed.	A priori sensitivity analyses for the logistic-regression sample-size calculation were performed using smaller hypothetical ORs of 9, 6, and 3 (Section 2.3, p. 5). No sensitivity analyses of the outcome models were performed.
**RESULTS**			
**Participants**	13a	Report participant numbers at each relevant stage of the study.	Section 2.4, pp. 5-6; Section 3.1, p. 7. Unique individuals invited/reached could not be determined; 308 responses were received, 8 incomplete responses were excluded, and 300 participants were analyzed.
**Participants**	13b	Give reasons for non-participation or exclusion at each stage, where known.	Reasons for non-participation could not be ascertained because the number of unique individuals reached was unknown; eight incomplete responses were excluded (Section 2.4, pp. 5-6).

Checklist item	No.	Reporting item	Where reported
**Participants**	13c	Consider presenting participant flow in a diagram.	No flow diagram was used; participant flow is reported in Section 2.4, pp. 5-6.
**Descriptive data**	14a	Report participant characteristics and information on relevant exposures/potential confounders.	Section 3.1; Table 1, p. 8
**Descriptive data**	14b	Indicate the number of participants with missing data for variables of interest.	Sections 2.4 and 2.6, pp. 5-7: eight incomplete responses were excluded; analysis-specific denominators and, where applicable, explicit option-level coding were used to account for conditional skip logic.
**Outcome data**	15	Report outcome event numbers or summary measures.	Sections 3.2-3.7, pp. 9-17; Tables 2-4; Figure 1; Supplementary Tables S3-S8
**Main results**	16a	Report unadjusted and adjusted estimates with precision and identify variables included in adjusted models.	Sections 3.4-3.6, pp. 11-17; Table 4; Supplementary Tables S4 and S7
**Main results**	16b	Report category boundaries when quantitative variables are categorized.	Table 1, p. 8; Supplementary File S2
**Main results**	16c	If relevant, translate relative measures into absolute risk over a meaningful time period.	Not applicable: cross-sectional odds ratios were not translated into absolute risks over time.
**Other analyses**	17	Report other analyses, including subgroup, interaction, or sensitivity analyses where applicable.	Secondary/exploratory analyses are reported in Sections 3.2-3.7 and Supplementary Tables S3-S8. No interaction analyses or outcome-model sensitivity analyses were performed. A priori sensitivity analyses for the logistic-regression sample-size calculation are described in Section 2.3, p. 5.
**DISCUSSION**			
**Key results**	18	Summarize key results in relation to the study objectives.	Discussion opening paragraph, p. 18; Conclusions p. 21
**Limitations**	19	Discuss limitations, including potential bias and imprecision and, where possible, their direction and magnitude.	Discussion limitations, pp. 20-21. Sources of bias and imprecision are discussed; their direction and magnitude were not separately quantifiable from the available data.
**Interpretation**	20	Provide a cautious overall interpretation considering objectives, limitations, multiplicity, and relevant evidence.	Discussion pp. 18-21
**Generalisability**	21	Discuss the generalisability (external validity) of the findings.	Discussion limitations, pp. 20-21; Conclusions p. 21
**OTHER INFORMATION**			
**Funding**	22	Report the source of funding and the role of funders, if any.	p. 22. This research received no external funding.

**Where the STROBE checklist denotes an item with an asterisk, information should be reported separately for relevant comparison groups where applicable.*

Note: Checklist items are based on the STROBE recommendations for cross-sectional studies [25].
