# Supplementary material for: Self-Perceived Knowledge and Self-Reported Clinical Use of Minimally Invasive Endodontics Among General Dentists and Endodontists in Türkiye: A Cross-Sectional Survey
Source: Healthcare (Basel). 2026 Oct 2;14(19):3279. doi: 10.3390/healthcare14193279 (PMC13665129; doi:10.3390/healthcare14193279)
Supplement: Supplementary file 1 [file healthcare-14-03279-s001.zip › Supplementary File S2.pdf]

Supplementary File S2. English Translation of the Questionnaire Administered to Participants

Participant Information and Electronic Informed Consent

Knowledge Levels and Clinical Practices of General Dentists and Endodontists Regarding Minimally Invasive Endodontic Approaches: A Survey Study

Dear Participant,

This survey is being conducted as part of a research study that received ethics committee approval (Decision No. 2025/372). The aim of the study is to evaluate the self-perceived knowledge, clinical practice patterns, and factors influencing the integration of minimally invasive endodontics into routine clinical practice among general dentists and endodontists actively practicing in Türkiye. The findings are expected to contribute to the development of minimally invasive endodontic practices and the planning of related educational processes in Türkiye.

Individuals who were actively practicing dentistry in Türkiye during the data collection period and whose professional status was general dentist or endodontist were eligible to participate. Dental students, individuals who had not yet graduated from dental school, those not actively practicing dentistry in Türkiye, and dental specialists working in specialties other than endodontics were outside the scope of the study.

Participation in this study is entirely voluntary. Refusing to participate or leaving the form before completing the questionnaire will not result in any loss of rights. Completion of the questionnaire takes approximately five minutes. No payment or incentive will be provided for participation. No significant foreseeable risk related to participation is expected, and no direct personal benefit to participants is anticipated.

Information obtained through the form will be protected in accordance with confidentiality principles and used only for scientific purposes. To restrict participation to one response per individual, the Google Forms "Limit to 1 response" setting was enabled; therefore, participants are required to sign in with a Google account. The "Collect email addresses" setting was disabled; accordingly, participants' email addresses are not collected as part of the research dataset. Only authorized members of the research team will have access to the research data. Participants may leave the form without providing a reason at any time before submitting the questionnaire. Because no direct identifiers are collected in the research dataset, after submission it will not be possible to distinguish a specific participant's response from the other responses and remove it from the dataset. Study results will be presented only in aggregate form in scientific publications, presentations, or reports, without information that would allow direct identification of participants.

To proceed to the questionnaire, participants must provide the electronic voluntary participation consent below. Only participants who electronically confirm that they have read the information provided, understood the conditions of participation, and voluntarily agree to participate may continue to the questionnaire. Individuals who do not provide voluntary consent will not be included in the study.

Electronic Informed Consent

"I have read and understood the information above. I have been informed about the purpose of the study, the conditions of participation, the voluntary nature of participation, and the use of my data. I declare that I meet the eligibility criteria for participation and voluntarily agree to participate in the study of my own free will."

• Yes, I have read the information above and voluntarily agree to participate in the study.

• No, I do not agree to participate in the study.

For participants who select "No," the survey will end at this stage.

Thank you for the time you devote to the questionnaire and for your contribution to our study.

If you have any questions about the study, you may contact the researchers below:

Principal investigator:

Asst. Prof. Melis Oya Ateş
Department of Endodontics, Faculty of Dentistry, Bolu Abant Izzet Baysal University, Bolu

melisoyaates@hotmail.com

Researcher:

Res. Asst. Selin Bıçaklıoğlu
Department of Endodontics, Faculty of Dentistry, Bolu Abant Izzet Baysal University, Bolu
selin.bicaklioglu@ibu.edu.tr

Section 1. Demographic and Professional Characteristics

1. What is your sex?
- Female
- Male

2. What is your age?
- 23-30
- 31-40
- 41-50
- 51 years or older

3. How many years have you been actively practicing dentistry?
- 0-5 years
- 6-10 years
- 11-15 years
- 16 years or more

4. What is your professional title?
- General dentist
- Endodontist

5. At which type of institution do you work?
- Public hospital/oral and dental health center
- University hospital
- Private practice/polyclinic
- Other .....

6. On average, how many teeth do you perform root canal treatment on per week?
- 0-5
- 6-10
- 11-15
- 16-20
- 21 or more

Section 2. Minimally Invasive Endodontics (MIE)

7. How knowledgeable are you about minimally invasive endodontic approaches?

- I have an adequate level of knowledge
- I have a moderate level of knowledge
- I have a low level of knowledge
- I have no knowledge

8. How adequate do you consider the education you received on minimally invasive endodontic approaches during your undergraduate/specialty training?

- Adequate
- Partially adequate
- Inadequate
- I received no training

9. Which sources do you prefer for obtaining information about minimally invasive endodontic approaches? (More than one option may be selected.)

- Congresses/seminars/courses
- Academic articles/publications
- Online education
- Social media platforms
- Sharing experiences with other dentists
- Other .....

10. How frequently do you use minimally invasive endodontic approaches in your clinical practice?

- Always
- Usually
- Sometimes
- Rarely
- Never

11. *If you do not use minimally invasive endodontic approaches, please do not answer this question.*

Which minimally invasive endodontic approaches do you use in your clinical practice? (More than one option may be selected.)

- Minimally invasive access cavity
- Vital pulp therapies
- Minimally invasive root canal preparation

12. *If you use the minimally invasive access cavity approach among minimally invasive endodontic approaches, please answer this question.*

When you use the minimally invasive access cavity technique, at which stage(s) do you experience difficulty? (More than one option may be selected; however, the "I do not experience any difficulty" option should not be selected together with any other option.)

- Access cavity preparation
- Gaining access to the root canals
- Root canal preparation
- Root canal irrigation
- Root canal obturation
- I do not experience any difficulty

13. *If you perform vital pulp therapies among minimally invasive endodontic approaches, please answer this question.*

When you perform vital pulp therapies, at which stage(s) do you experience difficulty? (More than one option may be selected; however, the "I do not experience any difficulty" option should not be selected together with any other option.)
- Caries removal
- Bleeding control
- Isolation
- Application of a pulp-protective agent
- Completion of the final restoration
- I do not experience any difficulty

14. *If you use the minimally invasive root canal preparation approach among minimally invasive endodontic approaches, please answer this question.*
When you perform minimally invasive root canal preparation, at which stage(s) do you experience difficulty? (More than one option may be selected; however, the "I do not experience any difficulty" option should not be selected together with any other option.)
- Root canal irrigation
- Root canal irrigation activation
- Root canal obturation
- I do not experience any difficulty

15. *If you do not use minimally invasive endodontic approaches, please do not answer this question.*
What are your reasons for preferring minimally invasive endodontic approaches in your treatments? (More than one option may be selected.)
- Faster healing
- Reduced loss of tooth structure
- Fewer complications
- Less postoperative pain
- Better patient cooperation
- Other .....

16. *If you use minimally invasive endodontic approaches, please do not answer this question.*
What are your reasons for not preferring minimally invasive endodontic approaches in your treatments? (More than one option may be selected.)
- Inadequate training/lack of knowledge
- High cost of the materials used
- Lack of necessary equipment
- Difficulty of application/technical sensitivity
- Insufficient time
- Other .....

17. Which radiographic diagnostic method(s) do you use before starting root canal treatment? (More than one option may be selected.)
- Panoramic radiography
- Periapical radiography
- Cone-beam computed tomography (CBCT)

18. Which magnification system(s) do you use during root canal treatment? (More than one option may be selected; however, the "I do not use any magnification system" option should not be selected together with any other option.)
- Dental loupes

- Dental operating microscope
- I do not use any magnification system

19. Do you use endodontic burs specifically designed for access cavity preparation or refinement?

- Yes
- No

20. Do you think that performing root canal treatments using minimally invasive endodontic approaches increases the success rate?

- Yes
- No
- Not sure

21. What do you think are the barriers to the routine clinical implementation of minimally invasive endodontic approaches? (More than one option may be selected.)

- Inadequate training/lack of knowledge
- Equipment cost
- Time required for implementation
- Difficulty changing established habits
- Technical difficulty/long learning curve
- Other .....

22. Which steps do you think should be prioritized to promote the wider adoption of minimally invasive endodontic approaches in Türkiye? (More than one option may be selected.)

- Greater emphasis on MIE in undergraduate/specialty education
- Increasing continuing education programs (courses, seminars, congresses, online education)
- Providing equipment support to clinics (microscopes, loupes, ultrasonic devices, dentin-preserving file systems, etc.)
- Translating current international publications into Turkish and making them more accessible
- Developing platforms for experience sharing among colleagues (online forums, case discussions)
- Other .....
