# Supplementary material for: Self-Perceived Knowledge and Self-Reported Clinical Use of Minimally Invasive Endodontics Among General Dentists and Endodontists in Türkiye: A Cross-Sectional Survey
Source: Healthcare (Basel). 2026 Oct 2;14(19):3279. doi: 10.3390/healthcare14193279 (PMC13665129; doi:10.3390/healthcare14193279)
Supplement: Supplementary file 1 [file healthcare-14-03279-s001.zip › Supplementary Table S1.pdf]

Supplementary Table S1. Comparison of self-perceived knowledge levels regarding minimally invasive endodontics (MIE) and the frequency of self-reported MIE use in clinical practice between general dentists and endodontists.

	**General dentists, *n* (%)**	**Endodontists, *n* (%)**	**Total, *n* (%)**	**Test statistic**	***p***	***adj. p***	**ES (95% CI)**
Self-perceived knowledge level regarding MIE approaches				100.042	<0.001[x]	<0.001	0.554 [0.445–0.669]
Adequate level of knowledge	25 (16.7)[a]	103 (68.7)[b]	128 (42.7)				
Moderate level of knowledge	88 (58.7)[a]	44 (29.3)[b]	132 (44.0)				
Low level of knowledge	35 (23.3)[a]	2 (1.3)[b]	37 (12.3)				
No knowledge	2 (1.3)	1 (0.7)	3 (1.0)				
Frequency of self-reported MIE use in clinical practice				40.668	<0.001[x]	<0.001	0.358 [0.255–0.473]
Always	8 (5.3)[a]	40 (26.7)[b]	48 (16.0)				
Usually	57 (38.0)	62 (41.3)	119 (39.7)				
Sometimes	45 (30.0)	31 (20.7)	76 (25.3)				
Rarely	19 (12.7)	14 (9.3)	33 (11.0)				
Never	21 (14.0)[a]	3 (2.0)[b]	24 (8.0)				

[x] Fisher's exact test with Monte Carlo simulation; a–b within each row, groups sharing the same superscript letter do not differ significantly; multiple comparisons were performed using the Bonferroni-adjusted Z test; adj. p = p value adjusted using the Benjamini–Hochberg false discovery rate (FDR) procedure; ES = effect size; Cramér's V; 95% confidence intervals were calculated using 2,000 bootstrap resamples; values are presented as frequency (percentage). MIE, minimally invasive endodontics; CI, confidence interval.
