# Supplementary material for: Self-Perceived Knowledge and Self-Reported Clinical Use of Minimally Invasive Endodontics Among General Dentists and Endodontists in Türkiye: A Cross-Sectional Survey
Source: Healthcare (Basel). 2026 Oct 2;14(19):3279. doi: 10.3390/healthcare14193279 (PMC13665129; doi:10.3390/healthcare14193279)
Supplement: Supplementary file 1 [file healthcare-14-03279-s001.zip › Supplementary Table S2.pdf]

Supplementary Table S2. Univariable and multivariable logistic regression analyses of factors associated with reporting difficulties during minimally invasive endodontic procedures

	No difficulty, *n* (%)	Difficulty reported, *n* (%)	Univariable model OR (95% CI)	*p*	Multivariable model OR (95% CI)	*p*
Difficulty during minimally invasive access cavity procedures						
Professional group						
General dentists	3 (11.1)	24 (88.9)	Reference	—	Reference	—
Endodontists	10 (23.8)	32 (76.2)	0.400 (0.099–1.613)	0.198	0.416 (0.061–2.812)	0.368
Professional experience in dentistry						
0–5 years	2 (8.7)	21 (91.3)	Reference	—	Reference	—
6–10 years	5 (23.8)	16 (76.2)	0.305 (0.052–1.779)	0.187	0.479 (0.069–3.323)	0.456
11–15 years	2 (22.2)	7 (77.8)	0.333 (0.039–2.829)	0.314	0.366 (0.024–5.618)	0.470
≥16 years	4 (25.0)	12 (75.0)	0.286 (0.045–1.798)	0.182	0.231 (0.027–2.004)	0.184
Institution of employment						
Public hospital/oral and dental health center	3 (25.0)	9 (75.0)	Reference	—	Reference	—
University hospital	4 (13.8)	25 (86.2)	2.083 (0.388–11.175)	0.392	1.249 (0.143–10.883)	0.840
Private practice/polyclinic	6 (21.4)	22 (78.6)	1.222 (0.250–5.982)	0.804	0.502 (0.046–5.504)	0.572
Weekly number of root canal treatments						
0–5	3 (14.3)	18 (85.7)	Reference	—	Reference	—
6–10	1 (5.9)	16 (94.1)	2.667 (0.251–28.278)	0.416	2.348 (0.176–31.398)	0.519
11–15	2 (18.2)	9 (81.8)	0.750 (0.106–5.324)	0.774	0.464 (0.048–4.490)	0.507
16–20	4 (36.4)	7 (63.6)	0.292 (0.052–1.650)	0.163	0.302 (0.038–2.432)	0.261
≥21	3 (33.3)	6 (66.7)	0.333 (0.053–2.115)	0.244	0.222 (0.015–3.324)	0.275
Difficulty during vital pulp therapies						
Professional group						
General dentists	18 (19.8)	73 (80.2)	6.551 (3.521–12.191)	<0.001	10.420 (3.993–27.195)	<0.001
Endodontists	84 (61.8)	52 (38.2)	Reference	—	Reference	—
Professional experience in dentistry						
0–5 years	28 (28.6)	70 (71.4)	4.375 (1.654–11.575)	0.003	4.349 (1.330–14.219)	0.015
6–10 years	44 (55.0)	36 (45.0)	1.432 (0.541–3.792)	0.470	3.036 (0.900–10.238)	0.073
11–15 years	16 (59.3)	11 (40.7)	1.203 (0.377–3.835)	0.755	3.520 (0.865–14.314)	0.079
≥16 years	14 (63.6)	8 (36.4)	Reference	—	Reference	—
Institution of employment						
Public hospital/oral and dental health center	19 (36.5)	33 (63.5)	1.737 (0.850–3.549)	0.130	2.756 (1.072–7.085)	0.035
University hospital	43 (45.3)	52 (54.7)	1.209 (0.666–2.195)	0.532	3.000 (1.270–7.083)	0.012
Private practice/polyclinic	40 (50.0)	40 (50.0)	Reference	—	Reference	—
Weekly number of root canal treatments						
0–5	19 (35.2)	35 (64.8)	3.132 (1.199–8.181)	0.020	1.343 (0.383–4.713)	0.645
6–10	26 (41.3)	37 (58.7)	2.419 (0.956–6.121)	0.062	1.044 (0.339–3.210)	0.940
11–15	18 (39.1)	28 (60.9)	2.644 (0.992–7.047)	0.052	1.905 (0.589–6.159)	0.282
16–20	22 (59.5)	15 (40.5)	1.159 (0.418–3.215)	0.777	0.786 (0.250–2.468)	0.680
≥21	17 (63.0)	10 (37.0)	Reference	—	Reference	—

	No difficulty, *n* (%)	Difficulty reported, *n* (%)	Univariable model OR (95% CI)	*p*	Multivariable model OR (95% CI)	*p*
Difficulty during minimally invasive root canal preparation						
Professional group						
General dentists	18 (21.4)	66 (78.6)	3.317 (1.763–6.244)	<0.001	2.379 (1.045–5.417)	0.039
Endodontists	57 (47.5)	63 (52.5)	Reference	—	Reference	—
Professional experience in dentistry						
0–5 years	23 (28.7)	57 (71.3)	1.126 (0.352–3.603)	0.841	1.345 (0.380–4.762)	0.646
6–10 years	34 (43.0)	45 (57.0)	0.602 (0.191–1.895)	0.385	0.896 (0.249–3.225)	0.867
11–15 years	13 (44.8)	16 (55.2)	0.559 (0.155–2.024)	0.376	0.854 (0.206–3.537)	0.827
≥16 years	5 (31.3)	11 (68.8)	Reference	—	Reference	—
Institution of employment						
Public hospital/oral and dental health center	14 (28.6)	35 (71.4)	1.480 (0.684–3.200)	0.320	2.651 (1.044–6.737)	0.040
University hospital	32 (41.6)	45 (58.4)	0.832 (0.437–1.587)	0.577	1.271 (0.590–2.740)	0.540
Private practice/polyclinic	29 (37.2)	49 (62.8)	Reference	—	Reference	—
Weekly number of root canal treatments						
0–5	12 (27.3)	32 (72.7)	3.556 (1.308–9.667)	0.013	3.034 (0.907–10.153)	0.072
6–10	19 (30.2)	44 (69.8)	3.088 (1.228–7.762)	0.017	3.082 (1.032–9.207)	0.044
11–15	16 (40.0)	24 (60.0)	2.000 (0.751–5.329)	0.166	2.114 (0.674–6.636)	0.199
16–20	12 (41.4)	17 (58.6)	1.889 (0.660–5.408)	0.236	1.866 (0.618–5.633)	0.269
≥21	16 (57.1)	12 (42.9)	Reference	—	Reference	—

OR, odds ratio; CI, confidence interval. Values are presented as *n* (%). Reference categories are indicated by "Reference." All multivariable models included professional group, professional experience in dentistry, institution of employment, and weekly number of root canal treatments simultaneously.

Model fit: minimally invasive access cavity difficulty, Cox & Snell $R^2 = 0.139$, Nagelkerke $R^2 = 0.224$, Hosmer–Lemeshow $\chi^2 = 6.336$, $p = 0.501$; vital pulp therapy difficulty, Cox & Snell $R^2 = 0.243$, Nagelkerke $R^2 = 0.325$, Hosmer–Lemeshow $\chi^2 = 6.365$, $p = 0.498$; minimally invasive root canal preparation difficulty, Cox & Snell $R^2 = 0.106$, Nagelkerke $R^2 = 0.145$, Hosmer–Lemeshow $\chi^2 = 7.707$, $p = 0.463$.
