# Supplementary material for: Self-Perceived Knowledge and Self-Reported Clinical Use of Minimally Invasive Endodontics Among General Dentists and Endodontists in Türkiye: A Cross-Sectional Survey
Source: Healthcare (Basel). 2026 Oct 2;14(19):3279. doi: 10.3390/healthcare14193279 (PMC13665129; doi:10.3390/healthcare14193279)
Supplement: Supplementary file 1 [file healthcare-14-03279-s001.zip › Supplementary Table S3.pdf]

Supplementary Table S3. Comparison of procedural stages at which difficulties were reported during minimally invasive endodontic procedures between general dentists and endodontists

Procedural stage	General dentists, *n* (%)	Endodontists, *n* (%)	Total, *n* (%)	Test statistic	*p*	adj. p	ES (95% CI)
Minimally invasive access cavity procedures*							
Access cavity preparation	2 (8.3)	5 (15.6)	7 (12.5)	—	0.686[x]	1.000	0.109 [0.000–0.289]
Gaining access to the root canals	18 (75.0)	22 (68.8)	40 (71.4)	0.046	0.831[y]	1.000	0.069 [0.000–0.234]
Root canal preparation	9 (37.5)	13 (40.6)	22 (39.3)	0.000	1.000[y]	1.000	0.032 [0.000–0.088]
Root canal irrigation	4 (16.7)	7 (21.9)	11 (19.6)	—	0.741[x]	1.000	0.065 [0.000–0.218]
Root canal obturation	14 (58.3)	19 (59.4)	33 (58.9)	0.000	1.000[y]	1.000	0.011 [0.000–0.027]
Vital pulp therapies*							
Caries removal	9 (12.3)	5 (9.6)	14 (11.2)	0.035	0.852[y]	0.852	0.042 [0.000–0.143]
Bleeding control	40 (54.8)	14 (26.9)	54 (43.2)	8.512	0.004[y]	0.010	0.277 [0.103–0.431]
Isolation	47 (64.4)	27 (51.9)	74 (59.2)	1.470	0.225[y]	0.281	0.125 [0.005–0.289]
Application of the pulp-protective agent	45 (61.6)	23 (44.2)	68 (54.4)	3.043	0.081[y]	0.135	0.172 [0.020–0.343]
Completion of the final restoration	11 (15.1)	22 (42.3)	33 (26.4)	10.237	0.001[y]	0.005	0.305 [0.122–0.459]
Minimally invasive root canal preparation*							
Root canal irrigation	41 (62.1)	45 (71.4)	86 (66.7)	0.873	0.350[y]	0.525	0.099 [0.006–0.273]
Irrigant activation	20 (30.3)	22 (34.9)	42 (32.6)	0.138	0.710[y]	0.710	0.049 [0.000–0.177]
Root canal obturation	40 (60.6)	19 (30.2)	59 (45.7)	12.040	<0.001[t]	<0.001	0.306 [0.124–0.458]

* Multiple-response items; each response option was coded as a separate binary variable and analyzed separately between professional groups using an option-specific 2 × 2 contingency table. [x] Fisher's exact test; [y] Yates-corrected Pearson's chi-square test; [t] Pearson's chi-square test; the Benjamini–Hochberg false discovery rate (FDR) procedure was applied across the option-specific comparisons within each multiple-response item, and adjusted p values are presented as adj. p. ES, effect size; CI, confidence interval. Phi was used as the effect-size measure for the 2 × 2 comparisons. The 95% CIs were calculated using the percentile bootstrap method with 2,000 resamples. Values are presented as *n* (%). Percentages were calculated among participants who reported at least one difficulty during the corresponding procedure: minimally invasive access cavity procedures, general dentists *n* = 24 and endodontists *n* = 32; vital pulp therapies, general dentists *n* = 73 and endodontists *n* = 52; minimally invasive root canal preparation, general dentists *n* = 66 and endodontists *n* = 63.
