# Supplementary material for: Self-Perceived Knowledge and Self-Reported Clinical Use of Minimally Invasive Endodontics Among General Dentists and Endodontists in Türkiye: A Cross-Sectional Survey
Source: Healthcare (Basel). 2026 Oct 2;14(19):3279. doi: 10.3390/healthcare14193279 (PMC13665129; doi:10.3390/healthcare14193279)
Supplement: Supplementary file 1 [file healthcare-14-03279-s001.zip › Supplementary Table S4.pdf]

Supplementary Table S4. Comparison of reported reasons for preferring and not preferring minimally invasive endodontics (MIE) approaches, radiographic diagnostic methods, and magnification systems used between general dentists and endodontists.

	**General dentists, *n* (%)**	**Endodontists, *n* (%)**	**Total, *n* (%)**	**Test statistic**	***p***	**adj. p**	**ES (95% CI)**
Reasons for preferring MIE approaches (general dentists, *n* = 150; endodontists, *n* = 150) *							
Faster healing time	29 (19.5)	50 (33.3)	79 (26.3)	7.578	0.006[t]	0.019	0.159 [0.045–0.271]
Less loss of tooth structure	118 (78.7)	141 (94.0)	259 (86.3)	13.674	<0.001[y]	<0.001	0.223 [0.118–0.318]
Fewer complications	48 (32.0)	70 (46.7)	118 (39.3)	6.761	0.009[t]	0.019	0.150 [0.037–0.260]
Less postoperative pain	27 (18.0)	40 (26.7)	67 (22.3)	3.248	0.072[t]	0.098	0.104 [0.008–0.211]
Better patient cooperation	38 (25.3)	45 (30.0)	83 (27.7)	0.816	0.366[t]	0.422	0.052 [0.002–0.156]
Reasons for not preferring MIE approaches (general dentists, *n* = 150; endodontists, *n* = 150) *							
Inadequate training/lack of knowledge	11 (7.3)	1 (0.7)	12 (4.0)	7.031	0.008[y]	0.019	0.170 [0.076–0.237]
High cost of materials	6 (4.0)	1 (0.7)	7 (2.3)	—	0.121[x]	0.151	0.110 [0.006–0.178]
Lack of necessary equipment	10 (6.7)	2 (1.3)	12 (4.0)	4.254	0.039[y]	0.065	0.136 [0.025–0.213]
Difficulty of application/technical sensitivity	13 (8.7)	2 (1.3)	15 (5.0)	7.018	0.008[y]	0.019	0.168 [0.068–0.241]
Insufficient time	12 (8.0)	2 (1.3)	14 (4.7)	6.069	0.014[y]	0.026	0.158 [0.051–0.231]
Radiographic diagnostic methods used before initiating root canal treatment (general dentists, *n* = 150; endodontists, *n* = 150) *							
Panoramic radiography	129 (86.0)	130 (86.7)	259 (86.3)	—	1.000[y]	1.000	0.010 [0.000–0.027]
Periapical radiography	117 (78.0)	148 (98.7)	265 (88.3)	29.111	<0.001[y]	<0.001	0.322 [0.244–0.393]
CBCT	12 (8.0)	35 (23.3)	47 (15.7)	12.211	<0.001[y]	<0.001	0.211 [0.091–0.310]
Magnification systems used during root canal treatment (general dentists, *n* = 26; endodontists, *n* = 96) *							
Dental loupe	23 (88.5)	94 (97.9)	117 (95.9)	—	0.064[x]	0.096	0.195 [0.026–0.429]
Dental operating microscope	6 (23.1)	28 (29.2)	34 (27.9)	0.135	0.713[y]	0.764	0.056 [0.001–0.167]

* Multiple-response items; each response option was coded as a separate binary variable and analyzed separately between professional groups using an option-specific 2 × 2 contingency table. [t] Pearson's chi-square test; [x] Fisher's exact test; [y] Yates-corrected Pearson's chi-square test. The Benjamini–Hochberg false discovery rate (FDR) procedure was applied across the option-specific comparisons reported in the table, and adjusted p values are presented as adj. p. ES, effect size; CI, confidence interval. Phi was used as the effect-size measure for the 2 × 2 comparisons. The 95% CIs were calculated using the percentile bootstrap method with 2,000 resamples. Values are presented as *n* (%). For the conditionally displayed items regarding reasons for preferring and not preferring MIE, participants who were not eligible to answer the corresponding item under the survey skip logic were coded as 0 (option not selected) for the option-specific binary analyses. Accordingly, these comparisons were based on the full professional-group samples (general dentists, *n* = 150; endodontists, *n* = 150), and the percentages represent full-group option-endorsement proportions rather than conditional proportions among MIE users or non-users. Radiographic diagnostic methods were likewise analyzed using the full professional-group samples (general dentists, *n* = 150; endodontists, *n* = 150). Magnification system types were evaluated among participants who reported using magnification systems (general dentists, *n* = 26; endodontists, *n* = 96).

MIE, minimally invasive endodontics; CBCT, cone-beam computed tomography.
