# Supplementary material for: Self-Perceived Knowledge and Self-Reported Clinical Use of Minimally Invasive Endodontics Among General Dentists and Endodontists in Türkiye: A Cross-Sectional Survey
Source: Healthcare (Basel). 2026 Oct 2;14(19):3279. doi: 10.3390/healthcare14193279 (PMC13665129; doi:10.3390/healthcare14193279)
Supplement: Supplementary file 1 [file healthcare-14-03279-s001.zip › Supplementary Table S5.pdf]

**Supplementary Table S5.** Univariable and multivariable logistic regression analyses of factors associated with the use of magnification systems and specialized endodontic burs for access cavity preparation or refinement.

| | **Not used, *n* (%)** | **Used, *n* (%)** | **Univariable model OR (95% CI)** | ***p*** | **Multivariable model OR (95% CI)** | ***p*** |
|---|---|---|---|---|---|---|
| Use of magnification systems during root canal treatment | | | | | | |
| Professional group | | | | | | |
| General dentists | 124 (82.7) | 26 (17.3) | Reference | — | Reference | — |
| Endodontists | 54 (36.0) | 96 (64.0) | 8.479 (4.949–14.526) | <0.001 | 9.131 (4.057–20.554) | <0.001 |
| Professional experience in dentistry | | | | | | |
| 0–5 years | 95 (72.5) | 36 (27.5) | Reference | — | Reference | — |
| 6–10 years | 50 (49.0) | 52 (51.0) | 2.744 (1.590–4.737) | <0.001 | 2.173 (1.025–4.608) | 0.043 |
| 11–15 years | 16 (47.1) | 18 (52.9) | 2.969 (1.368–6.444) | 0.006 | 1.807 (0.644–5.071) | 0.261 |
| ≥16 years | 17 (51.5) | 16 (48.5) | 2.484 (1.135–5.435) | 0.023 | 2.893 (1.035–8.087) | 0.043 |
| Institution of employment | | | | | | |
| Public hospital/oral and dental health center | 80 (92.0) | 7 (8.0) | Reference | — | Reference | — |
| University hospital | 39 (36.8) | 67 (63.2) | 19.634 (8.246–46.749) | <0.001 | 15.975 (5.509–46.325) | <0.001 |
| Private practice/polyclinic | 59 (55.1) | 48 (44.9) | 9.298 (3.929–22.002) | <0.001 | 18.610 (6.333–54.683) | <0.001 |
| Weekly number of root canal treatments | | | | | | |
| 0–5 | 52 (65.0) | 28 (35.0) | Reference | — | Reference | — |
| 6–10 | 60 (65.2) | 32 (34.8) | 0.990 (0.528–1.857) | 0.976 | 0.805 (0.341–1.901) | 0.621 |
| 11–15 | 25 (45.5) | 30 (54.5) | 2.229 (1.104–4.497) | **0.025** | 0.891 (0.344–2.307) | 0.811 |
| 16–20 | 19 (47.5) | 21 (52.5) | 2.053 (0.948–4.443) | 0.068 | 1.352 (0.462–3.961) | 0.582 |
| ≥21 | 22 (66.7) | 11 (33.3) | 0.929 (0.394–2.188) | 0.865 | 0.579 (0.171–1.967) | 0.381 |
| Use of specialized endodontic burs for access cavity preparation or refinement | | | | | | |
| Professional group | | | | | | |
| General dentists | 116 (77.3) | 34 (22.7) | Reference | — | Reference | — |
| Endodontists | 41 (27.3) | 109 (72.7) | 9.070 (5.369–15.324) | <0.001 | 5.970 (2.794–12.755) | <0.001 |
| Professional experience in dentistry | | | | | | |
| 0–5 years | 84 (64.1) | 47 (35.9) | Reference | — | Reference | — |
| 6–10 years | 44 (43.1) | 58 (56.9) | 2.356 (1.387–4.003) | 0.002 | 1.451 (0.697–3.023) | 0.320 |
| 11–15 years | 16 (47.1) | 18 (52.9) | 2.011 (0.938–4.309) | 0.072 | 0.932 (0.342–2.542) | 0.891 |
| ≥16 years | 13 (39.4) | 20 (60.6) | 2.750 (1.255–6.024) | 0.011 | 4.229 (1.541–11.600) | 0.005 |
| Institution of employment | | | | | | |
| Public hospital/oral and dental health center | 67 (77.0) | 20 (23.0) | Reference | — | Reference | — |
| University hospital | 31 (29.2) | 75 (70.8) | 8.105 (4.224–15.550) | <0.001 | 10.282 (4.081–25.903) | <0.001 |
| Private practice/polyclinic | 59 (55.1) | 48 (44.9) | 2.725 (1.454–5.108) | 0.002 | 5.928 (2.447–14.363) | <0.001 |
| Weekly number of root canal treatments | | | | | | |
| 0–5 | 48 (60.0) | 32 (40.0) | Reference | — | Reference | — |
| 6–10 | 61 (66.3) | 31 (33.7) | 0.762 (0.409–1.420) | 0.392 | 0.729 (0.322–1.650) | 0.448 |
| 11–15 | 24 (43.6) | 31 (56.4) | 1.937 (0.966–3.885) | 0.062 | 0.868 (0.350–2.149) | 0.759 |
| 16–20 | 14 (35.0) | 26 (65.0) | 2.786 (1.266–6.131) | 0.011 | 2.174 (0.783–6.037) | 0.136 |
| ≥21 | 10 (30.3) | 23 (69.7) | 3.450 (1.450–8.208) | 0.005 | 3.701 (1.083–12.644) | 0.037 |

OR, odds ratio; CI, confidence interval. Values are presented as *n* (%). Reference categories are indicated by "Reference." All multivariable models included professional group, professional experience in dentistry, institution of employment, and weekly number of root canal treatments simultaneously. Model fit: magnification system use, Cox & Snell $R^2$ = 0.364, Nagelkerke $R^2$ = 0.491, Hosmer–Lemeshow $\chi^2$ = 4.373, p = 0.822; specialized endodontic bur use for access cavity preparation or refinement, Cox & Snell $R^2$ = 0.333, Nagelkerke $R^2$ = 0.444, Hosmer–Lemeshow $\chi^2$ = 20.183, p = 0.010.
