# Supplementary material for: Self-Perceived Knowledge and Self-Reported Clinical Use of Minimally Invasive Endodontics Among General Dentists and Endodontists in Türkiye: A Cross-Sectional Survey
Source: Healthcare (Basel). 2026 Oct 2;14(19):3279. doi: 10.3390/healthcare14193279 (PMC13665129; doi:10.3390/healthcare14193279)
Supplement: Supplementary file 1 [file healthcare-14-03279-s001.zip › Supplementary Table S6.pdf]

Supplementary Table S6. Perceptions regarding minimally invasive endodontics (MIE), perceived barriers to routine clinical implementation, and recommendations for wider adoption among general dentists and endodontists.

	**General dentists, *n* (%)**	**Endodontists, *n* (%)**	**Total, *n* (%)**	**Test statistic**	***p***	***adj. p***	**ES (95% CI)**
Do you think performing root canal treatments using minimally invasive endodontic approaches increases the success rate?				3.016	0.211[t]	0.541	0.100 [0.082–0.192]
Yes	68 (45.3)	83 (55.3)	151 (50.3)				
No	20 (13.3)	17 (11.3)	37 (12.3)				
Not sure	62 (41.3)	50 (33.3)	112 (37.3)				
Perceived barriers to the routine clinical implementation of MIE *							
Lack of adequate training/knowledge	101 (67.3)	103 (68.7)	204 (68.0)	0.061	0.805[t]	0.805	0.014 [0.000–0.048]
Equipment cost	79 (52.7)	83 (55.3)	162 (54.0)	0.215	0.643[t]	0.786	0.027 [0.000–0.093]
Time-consuming application	74 (49.3)	58 (38.7)	132 (44.0)	3.463	0.063[t]	0.347	0.107 [0.013–0.227]
Difficulty changing established habits	85 (56.7)	88 (58.7)	173 (57.7)	0.123	0.726[t]	0.799	0.020 [0.000–0.064]
Technical difficulty/long learning curve	78 (52.0)	82 (54.7)	160 (53.3)	0.214	0.643[t]	0.786	0.027 [0.000–0.094]
Recommended measures to promote the wider adoption of MIE in Türkiye *							
Greater emphasis in undergraduate/specialty education	125 (83.3)	131 (87.3)	256 (85.3)	0.666	0.415[y]	0.761	0.057 [0.002–0.158]
Increase in continuing education programs	90 (60.0)	100 (66.7)	190 (63.3)	1.435	0.231[t]	0.541	0.069 [0.004–0.179]
Provision of equipment support to clinics	106 (70.7)	123 (82.0)	229 (76.3)	5.332	0.021[t]	0.231	0.133 [0.025–0.241]
Translation of current international publications into Turkish and improved accessibility	68 (45.3)	64 (42.7)	132 (44.0)	0.217	0.642[t]	0.786	0.027 [0.000–0.093]
Development of peer experience-sharing platforms	88 (58.7)	78 (52.0)	166 (55.3)	1.349	0.246[t]	0.541	0.067 [0.003–0.179]

* Multiple-response items; each response option was coded as a separate binary variable and analyzed separately between professional groups using an option-specific 2 × 2 contingency table. [t] Pearson's chi-square test; [y] Yates-corrected Pearson's chi-square test. The Benjamini-Hochberg false discovery rate (FDR) procedure was applied across the comparisons reported in the table, and adjusted p values are presented as adj. p. ES, effect size; CI, confidence interval. Cramér's V was used for the 2 × 3 comparison of perceptions regarding treatment success, whereas Phi was used for the 2 × 2 option-specific comparisons. The 95% CIs were calculated using the percentile bootstrap method with 2,000 resamples. Values are presented as *n* (%). MIE, minimally invasive endodontics.
